# Identification of a Potential Ovarian Cancer Stem Cell Gene Expression Profile from Advanced Stage Papillary Serous Ovarian Cancer

**DOI:** 10.1371/journal.pone.0029079

**Published:** 2012-01-17

**Authors:** Vinod Vathipadiekal, Deepa Saxena, Samuel C. Mok, Peter V. Hauschka, Laurent Ozbun, Michael J. Birrer

**Affiliations:** 1 Massachusetts General Hospital Cancer Center, Massachusetts General Hospital, Harvard Medical School, Boston, Massachusetts, United States of America; 2 Department of Orthopaedic Surgery, Children's Hospital Boston, Harvard Medical School, Boston, Massachusetts, United States of America; 3 Department of Gynecologic Oncology, The University of Texas M. D. Anderson Cancer Center, Houston, Texas, United States of America; 4 Cell and Cancer Biology Branch, Center for Cancer Research, National Cancer Institute, National Institutes of Health, Bethesda, Maryland, United States of America; The University of Hong Kong, China

## Abstract

Identification of gene expression profiles of cancer stem cells may have significant implications in the understanding of tumor biology and for the design of novel treatments targeted toward these cells. Here we report a potential ovarian cancer stem cell gene expression profile from isolated side population of fresh ascites obtained from women with high-grade advanced stage papillary serous ovarian adenocarcinoma. Affymetrix U133 Plus 2.0 microarrays were used to interrogate the differentially expressed genes between side population (SP) and main population (MP), and the results were analyzed by paired T-test using BRB-ArrayTools. We identified 138 up-regulated and 302 down-regulated genes that were differentially expressed between all 10 SP/MP pairs. Microarray data was validated using qRT-PCR and17/19 (89.5%) genes showed robust correlations between microarray and qRT-PCR expression data. The Pathway Studio analysis identified several genes involved in cell survival, differentiation, proliferation, and apoptosis which are unique to SP cells and a mechanism for the activation of Notch signaling is identified. To validate these findings, we have identified and isolated SP cells enriched for cancer stem cells from human ovarian cancer cell lines. The SP populations were having a higher colony forming efficiency in comparison to its MP counterpart and also capable of sustained expansion and differentiation in to SP and MP phenotypes. 50,000 SP cells produced tumor in nude mice whereas the same number of MP cells failed to give any tumor at 8 weeks after injection. The SP cells demonstrated a dose dependent sensitivity to specific γ-secretase inhibitors implicating the role of Notch signaling pathway in SP cell survival. Further the generated SP gene list was found to be enriched in recurrent ovarian cancer tumors.

## Introduction

Epithelial ovarian cancer is the fifth leading cause of death in women in the United States. In 2010, there will be an estimated 21,880 new cases and 13,850 deaths from ovarian caner in the United States [1]. Although the 5-year survival rate is >90% for women with early-stage ovarian cancer, about 80% of women present with late-stage disease and have a 5-year survival rate of only 30%. Standard therapy includes cytoreductive surgery with first-line combination chemotherapy [2]. 75% of patients initially respond to conventional chemotherapy, however, >80% of these women eventually relapse and die from chemotherapy resistant disease [2].

There is increasing evidence that small populations of cells within tumors called cancer stem cells (CSC) contributes to tumor maintenance and progression and are intrinsically resistant to therapies designed to destroy rapidly dividing cells [3], [4], [5], [6], [7]. CSC have been described from several human solid cancers, such as breast [8], brain [9], [10], colon [11], [12], head and neck [13], and pancreatic cancer[14]. Experiments performed on human acute myeloid leukemia [15] and solid tumors [8], [9] show that CSCs display three functional characteristics: 1) they have the tumorigenic potential to form tumors when injected into nude mice, 2) they express distinct surface markers allowing for reproducible and differential purification, and 3) they have the ability to recreate the full phenotypic heterogeneity of the parent tumor [16], [17]. Thus the definition for CSC is a functional one and shares two important functional characteristics with normal stem cells: self-renewal and differentiation [3], [7].

The difficulty in characterizing normal and cancer stem cells is, these cell populations are rare and the absence of specific cell surface markers represents a challenge to isolate and identify pure stem cell populations. The inability to isolate a pure stem cell population has created intense debate about the CSC model [18], [19], [20]. Several stem cell markers (CD133, CD44, Sca1) have been used successfully to isolate stem cells in normal and tumor tissue [21], [22], [23]. However, no marker has been identified that is exclusively present on stem cells [7]. Cell surface markers found on stem cells from one tissue are not always useful for identifying stem cells from another tissue since many of these markers are also found on non-stem cells from unrelated tissues and organs [7].

Goodell et al first reported a small population of cells showing a distinct FACS profile off to the side of the main population due to a more efficient Hoechst dye efflux and lower fluorescent intensity signal [24]. This subset of cells is referred to as the side population (SP) and is enriched for hematopoietic stem cells from murine bone marrow [24]. Many studies of SP have been performed in a number of cancers such as leukemias, brain, prostate, GI tract, melanoma, retinoblastoma, and many cancer cell lines, leading to the hypothesize that the SP is enriched with CSC [8], [25], [26], [27], [28], [29], [30], [31]. It has now been shown that ovarian cancer, like many other tumors, contains SP cells that apparently correspond to the CSCs responsible for the tumor growth [32], [33]. SP cells from ovarian cancer have been demonstrated for its sensitiveness towards mullerian inhibiting substance and IFN-α [32], [33]. We propose the side population of ascites from women with high-grade advanced stage papillary serous ovarian adenocarcinoma would be enriched for cancer stem-like cells, and would express a gene signature trend for “stemness” in ovarian cancer stem-like cells.

## Materials and Methods

### Ethics Statement

Fresh ascites was obtained from 10 women with high-grade advanced stage ovarian adenocarcinoma at the time of primary cytoreductive surgery at Brigham and Women's Hospital, Boston, MA. All the specimens were collected under the protocols approved by the institutional review boards of the Brigham and Women's Hospital and were obtained with informed written consent from the patients. Animal care and experiments were carried out in accordance with the guidelines and approval by the MGH Animal Care and Use Committee (Protocol no. 2009N000148).

### Tumor specimen and isolation of sub-populations of cells

Fresh ascites was obtained from 10 women with high-grade advanced stage ovarian adenocarcinoma at the time of primary cytoreductive surgery at Brigham and Women's Hospital, Boston, MA. The ascites samples were centrifuged at 1400 RPM to isolate the cells. After lysis of the erythrocytes, the remaining cells were seeded and grown in culture dishes without passaging. Before sorting, cells were stained with CD45 to rule out contamination with blood cells and with CA125 antibody to confirm the ovarian origin of these cells. CD 45 and CA 125 analysis were carried out using FACS. CD45 and CA125 antibodies were purchased from BioLegend and Invitrogen respectively. Between day 7 and 10 cells were stained with Hoechst33342 or 0.5 µg/mL Rhodamine 123 (Rho) [34] and sorted on a BD FACSAria equipped with a violet laser. Control experiments verified that the Hoechst^DIM^ *vs.* Rho^DIM^ side population (SP) fractions isolated by both methods were similar by % abundance and gene expression profile.

### Affymetrix genechip hybridization and image acquisition

Total RNA was extracted from the SP and MP using the RNeasy kit (Qiagen, Germantown, MD). Total RNA quality was first checked by BioAnalyzer (Agilent, Palo Alto, CA) before further manipulation. Two rounds of amplification were used as previously described [35]. Hybridization, processing and image acquisition was done as described previously [35].

### Data normalization and analysis

All array data is Minimum Information About a Microarray Experiment (MIAME) compliant and the raw data has been deposited in a MIAME compliant database (GEO, Accession Number: Series GSE33874)

Genechip images and data sets were uploaded into the National Cancer Institute's Microarray Analysis Database (mAdb) for evaluation (http://nciarray.nci.nih.gov/index.shtml). Low-level analysis included array normalization and estimation of expression level. This was accomplished by invariant set normalization to adjust the overall signal level of the arrays to the same level for further comparison [36]. Next, we applied a model-based approach to calculate the gene expression level. The low-level analysis was conducted using MAS5.0 normalized data in BRB ArrayTools version 3.6.0 software designed by Dr. Richard Simon and Amy Peng Lam of the Biometrics Research Branch, NCI, NIH.

### Quantitative real-time PCR

qRT-PCR was performed on 50 ng of amplified RNA using an iCycler Real-Time Detection System (Bio-Rad Laboratories, Inc., Hercules, CA, USA) as previously described [37], using the SuperScript III Platinum SYBR Green One-Step qRT-PCR according to the manufacturer's instruction (Invitrogen, Carlsbad, CA). Relative expression levels of each gene were obtained by normalization to the expression levels of three housekeeping genes (*Cyclophilin, GUSB, GAPDH*) [38]. Real-time PCR expression values were used for correlation analyses with microarray signal intensities. Pearsons' and Spearmans' rank correlation was performed using GraphPad Prism 5.00 (GraphPad Software Inc., San Diego, CA).

### Cell lines and culture conditions

The human ovarian cancer cell lines SKOV3, A224, OVCAR-3, and UCI-107 were maintained in RPMI 1640 (Invitrogen Life Technologies) supplemented with 10% fetal bovine serum, 1% L-glutamine, and 1% penicillin/streptomycin in humidified incubator with 5% CO_2_ at 37°C as described by Mok et al [39].

### Flow cytometry analysis

The cells were detached by trypsinization, centrifuged and resuspended in tissue culture medium containing 2% serum at a concentration of 1×10^6^ cells/mL. The cells were labeled with 5.0 µg/mL Hoechst33342 dye at 37°C for 90 min either alone or in combination with ABCB1 efflux pump inhibitor Verapamil (100 µM). At the end of the incubation the cells were centrifuged in the cold and resuspended in cold fresh medium with 2% serum. 7-Amino-actinomycin D (7AAD) was added to the cells to a final concentration of 2 µg/mL prior to FACS analysis to exclude the dead cells from analysis. The SP analysis was done using a BD LSRII System (BD Biosciences). The Hoechst dye was excited with UV laser and its fluorescence was measured with both 675LP (Hoechst Red) and 440/40 filters (Hoechst Blue).

### Staining for putative stem cell markers

Immunophenotypic characterization of SP cells was carried out using fluorescent isothiocyanate (FITC), phycoerythrin (PE), and allophycocyanin (APC) conjugated monoclonal antibodies. Prior to antibody staining, the cells were stained for SP cells using Hoechst 33342 as described above. The cells were then washed and incubated with CD24-RPE (AbD Serotec), CD34-FITC (Miltenyi Biotec), CD44-FITC (AbD Serotec), CD117-PE (Miltenyi Biotec), and CD133-APC (Miltenyi Biotec) in PBS at 4°C for 15 min in the dark. The fluorescence compensation setting for multicolor flow cytometric analyses was performed using BD CompBeads Anti-Mouse Ig, κ (BD Biosciences) and all analysis was carried out using BD LSRII System (BD Biosciences). The FITC and PE antibodies were excited at 488 nm and collected using 530/30 nm (FITC), 575/26 nm (PE) filters. The APC antibody was excited at 633 nm and collected at 660/20 nm.

### Colony forming efficiency assay

For the analysis of colony forming efficiency (CFE), SP and MP cells were plated at 100 cells per well in a six well tissue culture plate and grown for 14 days. The cells were then fixed with cold methanol for 20 min at 4°C and stained with 0.5% crystal violet solution in order to count the number of colonies by microscopy. The analysis was performed for two subsequent passages. Colony forming efficiency was also calculated as the percentage of single cells that generated colonies at day 14. The cells were stained with Hoechst dye and single cells were sorted using FACS for MP and SP into a 96 well plate containing 200 µL of tissue culture medium. The cells were grown for 14 days before fixing and staining with methanol and crystal violet solution.

### Anchorage independent growth

Anchorage independent growth was examined by soft agar cloning. A 7% stock of low-gelling agarose in PBS was diluted in RPMI media/10% serum to a final concentration of 0.7% agarose. For the bottom layer, 1.5 mls of 0.7% agarose was added to 6-well plates and allowed to cool at 4°C. The leftover 0.7% agarose in media was further diluted in RPMI media/10% serum to a final agarose concentration of 0.35%. For the top layer, 2500 cells (SKOV3)/5000 cells (A224) were plated in 6 mls of 0.35% agarose. Following incubation for 1 hr at 4°C, the plates were transferred to 37°C and incubated for 14 days. The cells were then stained overnight with 0.5 mg/ml of nitroblue tetrazolium (Sigma-Aldrich, St. Louis, MO) and colonies between 100–2000 microns were counted with the Biocount 4000P (Biosys, Germany). Two independent experiments were performed with each sample plated in triplicate per experiment.

### Growth of SKOV SP and MP cells *in vivo*

Female athymic nude mice (Balb/C athymic mice) were purchased from the Charles River Laboratories (Wilmington MA, USA). The mice were used in these experiments when they were 4 to 6 weeks old. To produce tumors, SKOV3-SP and MP (5×10^4^ cells/100 µL PBS) cells were injected subcutaneously into the dorsal area of the mice. For *in vivo* injections, the cells were sorted into SP and MP fractions and cultured for 8 days. The cells were then trypsinized and centrifuged at 800 rpm for 7 minutes at 4°C, washed twice with PBS, and reconstituted in PBS (GIBCO/Invitrogen). Only single-cell suspensions with >95% viability, as determined by trypan blue exclusion, were used for the *in vivo* injections.

### Repopulation of SP and MP fractions by SKOV3 and A224 SP cells

The cells were stained with Hoechst 33342 dye as described above for sorting. The sorted SP cells and MP cells were cultured separately under the same conditions for 8 days before being restained with Hoechst 33342 dye and reanalyzed.

### Effect of γ-Secretase Inhibitor IX (GSI-IX) SKOV3 SP and MP cells

The γ-Secretase Inhibitors GSI-IX (Calbiochem, EMD Biosciences Inc., La Jolla, CA) was dissolved in 100% dimethyl sulphoxide (DMSO) to make the stock solutions (5 mg/ml), which was then diluted in culture medium to obtain the desired concentrations. The sorted SKOV3 SP and MP cells were treated with 10 and 20 µg of inhibitors to evaluate the effect of the inhibitor on colony forming efficiency of the cells. Control SKOV3 SP and MP cells were treated with DMSO in amounts equal to the concentration of DMSO necessary to solubilize the inhibitors.

### Enrichment of SP gene signature in recurrent ovarian cancer patients expression database

The enrichment of SP gene signature in recurrent ovarian cancer patients compared to the primary tumor was analyzed using the clinical expression databases of human ovarian cancer. Six patient's information's containing expression files for 12 samples (primary and recurrent) were available in our clinical database. We have obtained expression values for each probe set using the Robust Multichip Average method (RMA) of Biometric Research Branch (BRB) Array Tools. The probe sets scored as absent was excluded and only upregulated genes of SP cell gene signature were included in the analysis. qRT-PCR was carried out on primary and recurrent ovarian tumor samples using randomly selected genes from SP gene list.

## Results

### Expression profiling of SP/MP cells from ascites specimens

**Figure 1:**
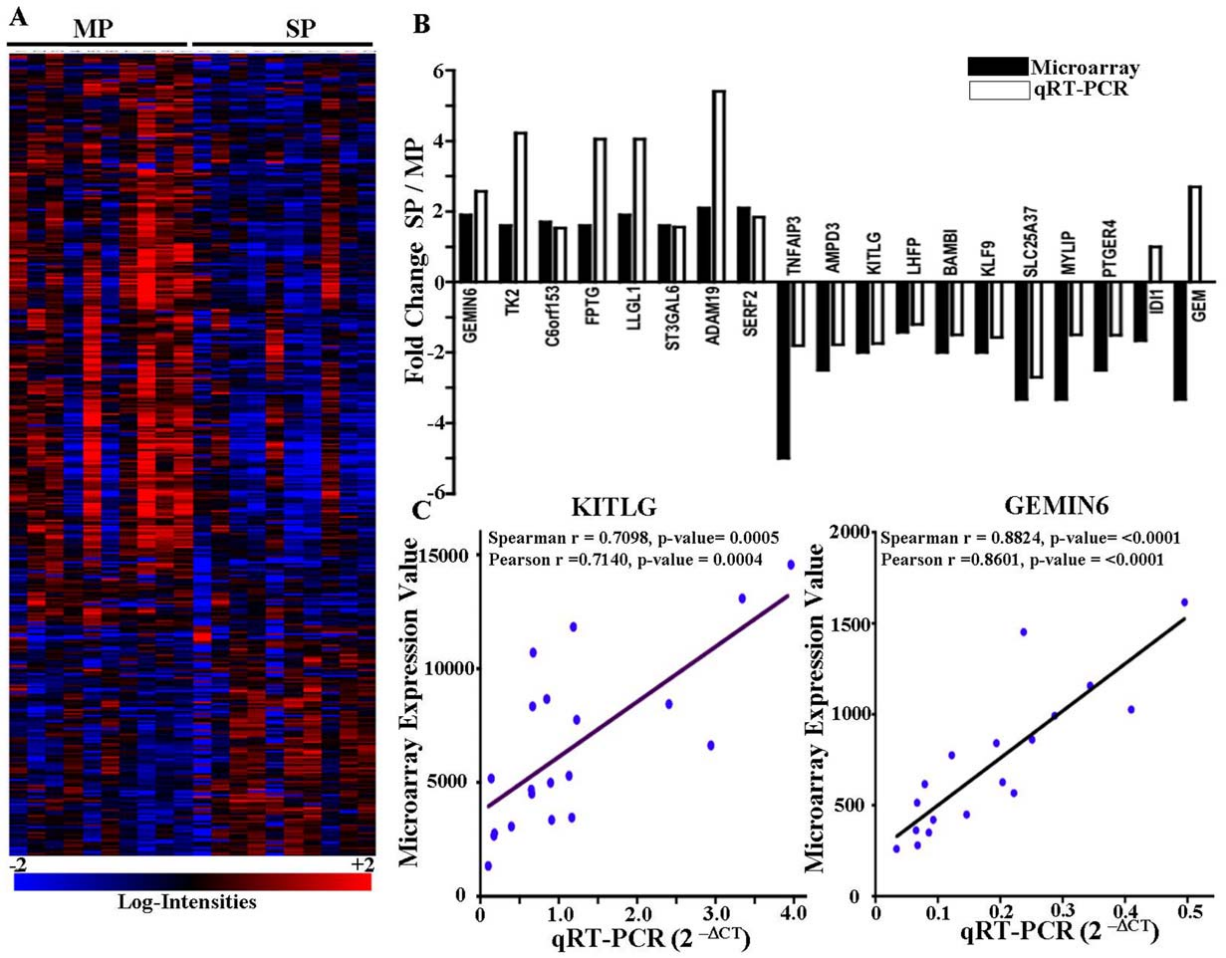
Expression profiling of SP/MP cells from ascites specimens. (A) Heatmap showing the expression pattern of 438 probesets that discriminated SP cells from MP cells in ovarian cancer patients. Vertical columns represent individual samples; horizontal rows represent individual genes. The red and blue color indicates up and down-regulation respectively. (B) Validation of microarray data using qRT-PCR: 19 randomly selected genes were used to validate the microarray data. To calculate the relative expression for each gene, the 2^−ΔΔCT^ method was used averaging the CT values for the three housekeeping genes (Cyclophilin A, GUSB, GAPDH) for a single reference gene value. (C) Representative plots for correlation analysis between the microarray and qRT-PCR data: The 2^−ΔCT^ values (qRT-PCR) were plotted against signal intensity values (Microarray). The correlation analysis was performed for each gene by Pearson's and Spearman's method using GraphPad Prism version 4.00.

The positive CA 125 staining using FACS confirmed the ovarian origin of cells isolated from ascites (Figure S1) and these cells were analyzed for SP and MP cells using Hoechst and/or Rho staining. A representative graph for the SP and MP sort from ascites sample is shown in Figure S2, and in all patient samples the SP ranged around 0.25% (Table 1). Ascites specimens were not examined without culture, and to rule out contamination with blood cells, we used only cells that were negative for CD45 in our analysis. Isolated SP and MP subpopulations were subcultured and global gene expression profiles using the Affymetrix U133 Plus 2 arrays were obtained. An informative data set of 16964 sequences was generated after initial filtering of the data. 446 differentially expressed probesets representing 432 genes were identified by paired T-test analysis at a significance of *P*<0.01. 142 probesets were up-regulated, and 304 probesets were down-regulated, in the SP compared to the MP. A heatmap showing the 438 differentially expressed probesets for all SP and MP samples is shown in figure 1A.

**Table 1:** The percentage of the SP cells identified in ascites samples.

Patient ID's	Percentage SP identified
Sample 1	0.23
Sample 2	0.16
Sample 3	n.d.
Sample 4	0.2
Sample 5	0.2
Sample 6	0.3
Sample 7	n.d.
Sample 8	n.d.
Sample 9	0.3
Sample 10	n.d.

n.d. (not determined).

The microarray results were validated by performing qRT-PCR analysis on 19 randomly selected differentially expressed genes. The qRT-PCR expression differences for the over-expressed and under-expressed genes in the SP compared to the MP were validated for 17/19 genes (Figure 1B). The correlation of data generated with microarray and qRT-PCR were analyzed using Pearsons' and Spearmans' rank correlations analysis (Figure S3 & Table S1, S2). 17/19 genes showed significant correlations between microarray expression data and qRT-PCR expression data resulting in a microarray validation rate of 89.5%. Linear regression plots of two representative genes (KITLG and GEMIN6) are shown in figure 1C.

### Identification of signaling pathways contributing to ovarian cancer SP cell survival, self-renewal and tumor maintenance

**Figure 2:**
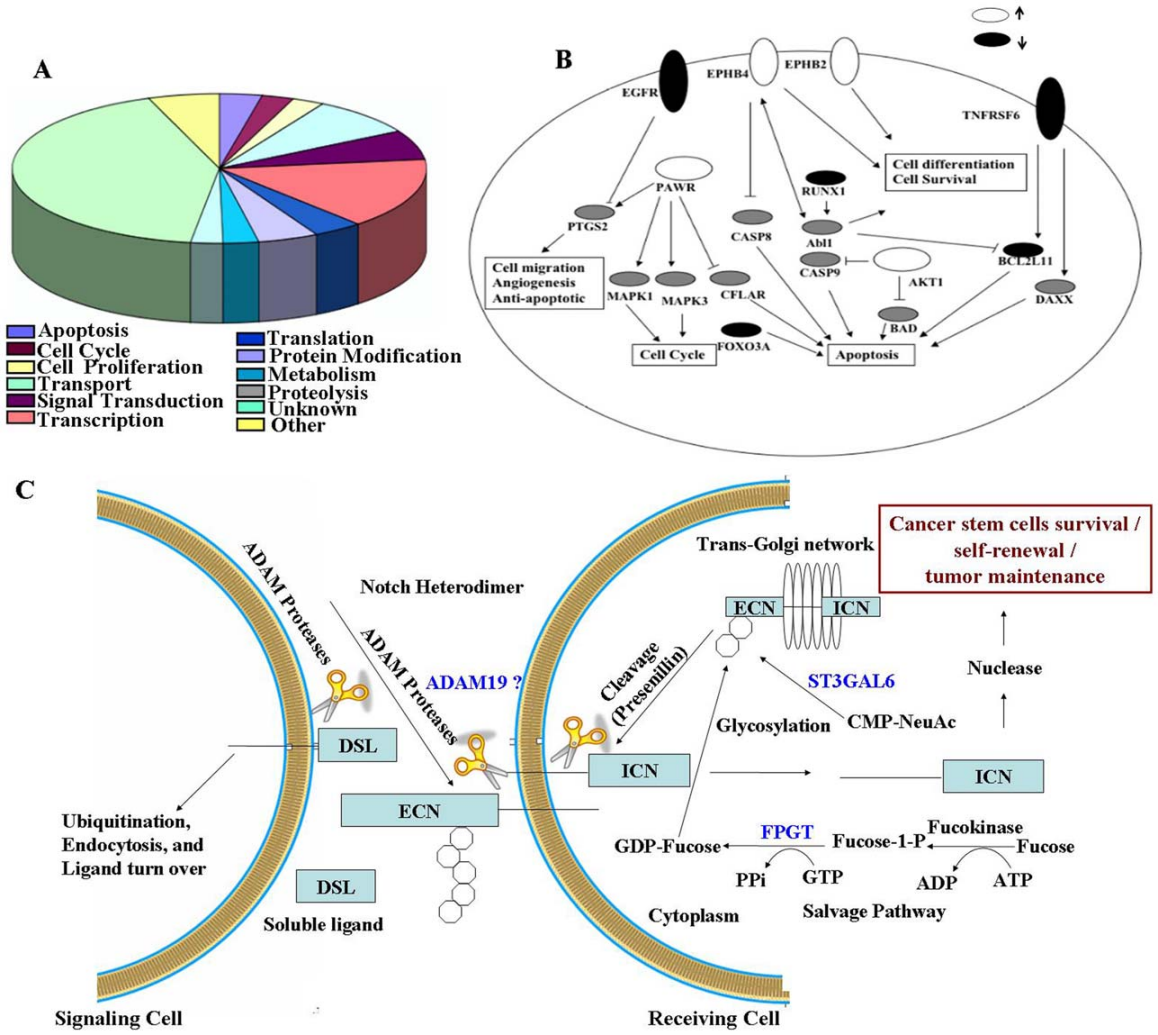
Identification of putative signaling events contributing to ovarian cancer SP cell survival, self-renewal and tumor maintenance. (A) Gene ontology analysis of microarray data (P value<0.01): The pie diagram shows the biological functions of the differentially expressed genes among SP and MP cells. The gene signature was enriched for genes in gene ontology biological processes of apoptosis, cell cycle, cell proliferation, transport, signal transduction, transcription, translation, protein modification, metabolism and proteolysis. Other represents genes involved in defense response, vasculogenesis, blood coagulation, visual perception, ontogenesis, cell matrix adhesion, and initiation of primordial ovarian follicle growth. (B) Graphical representation of the literature derived facts about the biological pathways involved in SP cells. Pathway Studio 6.0 software was used to identify the activated pathways in SP cells. Solid symbols representing the genes (EGFR, TNFRSF6, BCL2L11, FOXO3A, RUNX1) as down regulated in SP cells, the open symbols represents the upregulated genes (EPHB4, EPHB2, PAWR, AKT1) and gray symbols are the genes whose expression did not change significantly between SP and MP cells. (C) The Notch signaling pathway: The figure shows the schematic of Notch signal transduction elements. The overexpressed proteins in SP cells (FPTG, ST3GAL6 and ADAM19) are shown in blue color. Post-translational modification of precursor Notch-protein includes cleavage by proteases and glycosylations in the trans-Golgi. Adherence of Notch extracellular domain (ECN) with Notch intracellular domain (ICN) results in mature Notch heterodimers and they are transferred to the cell membrane. Receptor interaction with the ligands of the DSL family (Delta, Serrate, Lag3) on neighboring cells is modulated by glycosylations of ECN. Successful interaction of extracellular ligand regions with EGF-like repeats of ECN lead to the proteolytic cleavages of Notch transmembrane domain by two sequential steps by ADAM proteases and presenilins with γ–secretase activity. The released Notch intracellular domain is translocated to the nuclease where it interacts with CSL1, replacing CSL repressors and forming a transcription complex with Mastermind-like factors and transcriptional coactivators. This transcriptional complex activates downstream target genes and may account for cancer stem cell survival, self-renewal and tumor maintenance in ovary.

Of the 438 differentially regulated genes, slightly more were underexpressed (68%) in SP cells compared to MP cells. The differentially expressed gene signature was enriched for genes in Gene Ontology biological processes (*P*<0.01) of apoptosis, cell cycle, cell proliferation, transport, signal transduction, transcription, translation, protein modification, metabolism and proteolysis (Figure 2A). A major part of the genes were not assigned with any biological functions, and it may contain novel genes associated with potential stem-like cell characteristics. To identify signaling pathways associated with SP cell survival and differentiation the microarray data was analyzed using Pathway Studio 6.0 (Ariadne Genomics). Data mining for biologically relevant processes shows the biological processes associated with the differentially expressed genes (Figure 2B). Genes implicated in normal stem cell function NUP [40], ST3GAL [41], LTBP [42] were upregulated in SP. Apart from this; SP cells were demonstrating different genes associated with an active anti apoptosis mechanism (Figure 2B).

We have identified several genes that may impart stemness characteristics to ovarian SP cells. Due to the heterogeneity of the cell population in the SP, we anticipate that clear pathways may not be obviously present. ADAM19, FPGT and ST3GAL6 were found to be over expressed in SP cells and may be potential cancer stem-like cell related genes. Based on the expression profile of FPGT, ST3GAL6 and ADAM19 in SP gene signature list, we propose a probable mechanism, which may be active in the SP cells that accounts for the SP cell survival, differentiation and tumor maintenance in ovary (Figure 2C). The figure shows the involvement of three enzymes in activation of Notch signaling pathway. FPTG activates the synthesis of GDP-fucose and which was incorporated to the Notch receptors by fucosyl transferases. The enzyme ST3GAL6 can activate the addition of terminal sialic acid residue, further the proteolytic cleavages of Notch transmembrane domain on the outside of the cell was catalyzed by ADAM19.

### Identification of side population (SP) cells from ovarian cancer cell lines

**Figure 3:**
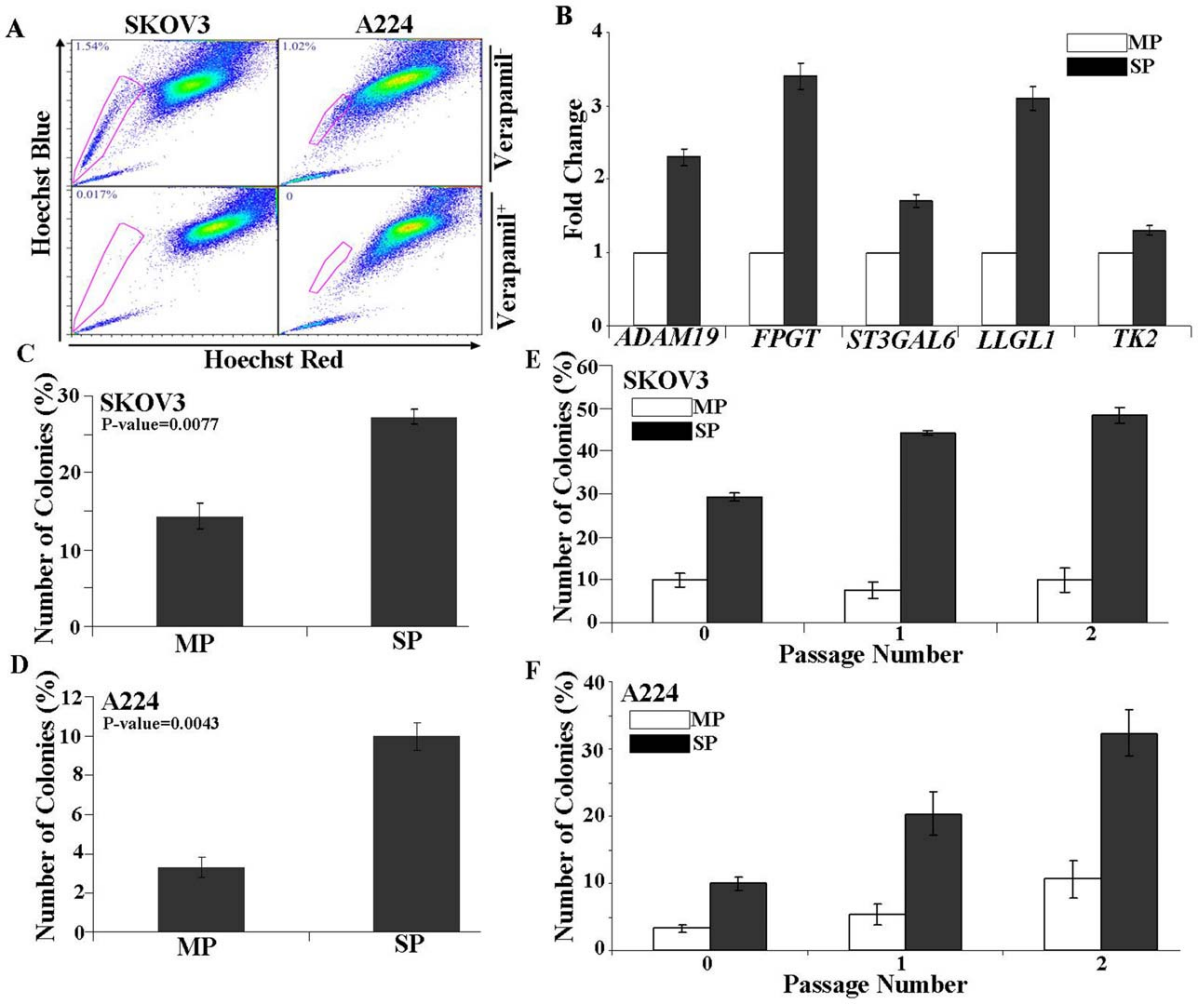
Identification and validation of side population cells from ovarian cancer cell lines. (A) Identification of SP cells in established human ovarian cancer cell lines. SKOV3 and A224 cell lines were labeled with Hoechst 33342 dye and analyzed by flow cytometry. The SP cells, which disappeared in the presence of Verapamil (a multidrug transporter inhibitor), are outlined and shown as the percentage of the total cell population. Similar results were obtained for three independent measurements. (B) Validation of randomly selected genes from the SP gene list on the SP and MP cells isolated from the SKOV3 cell lines. (C, D) Colony forming efficiency assay: Colony forming efficiency of sorted SP and MP cells from SKOV3 and A224 cell lines. For the analysis of colony forming efficiency (CFE), SP and MP cells were sorted and plated in equal numbers in tissue culture six well plates and grown for 14 days. The cells were then fixed with cold methanol and stained with 0.5% crystal violet solution to count the number of colonies by microscopy. The experiments were carried out in triplicates. (E, F) Passage Number: Colony forming efficiency of sorted SP and MP cells from SKOV3 and A224 cell lines were evaluated as a function of passage number. Cells were fixed with cold methanol and stained with 0.5% crystal violet.

We identified SP cells from four human ovarian cancer cell lines using flow cytometric analysis of the exclusion of DNA binding dye, Hoechst33342. As a control we used Verapamil, which blocks the activity of drug transporter proteins, preventing them from effluxing the dye. Figure 3A shows the fluorescent-activated cell sorting profile of SKOV3 and A224 ovarian cancer cell lines. The gated SP cells are outlined and shown as the percentage of the total cell population. The SP is ablated when verapamil is included in the Hoechst incubation. We detected SP cells in all four cancer cell lines evaluated, SKOV3 (1.54%), A224 (1.02%), OVCAR-3 (0.08%), and UCI-107 (0.12%). Further to check the authenticity of side population cells isolated, qRT-PCR was carried out on randomly selected genes from patient SP gene list (ADAM19, FPGT, ST3GAL6, LLGL1 and TK2). All genes were well validated on SP cells isolated from SKOV3 supporting the enrichment of cancer stem-like cells in isolated cells of side populations (Figure 3B). The over expression of ADAM 19 and FPGT in SP cells were validated at protein level using immunoflourescence and western blotting methods respectively (Figure S4). To validate the Notch pathway genes identified using pathway studio, we evaluated the Notch target genes using qRT-PCR in the SP and MP populations. A significant upregulation of Notch target genes including *HES1* and *NOTCH1* was detected in SP population in compared to MP counterpart (Figure S5).

### Biological validation of SP cells isolated from cell lines

The colony forming efficiency (CFE) of SP and MP cells isolated from SKOV3 and A224 cell lines were evaluated. SKOV3 MP cells had a CFE of 13.0±2.0% compared to a CFE of 27±1.0% for the SP cells (*P = 0.0077*) (Figure 3C). A224 MP cells showed a CFE of 3.0±1.0% compared to a CFE of 9.0±1.0% for the SP cells (*P = 0.0043*) (Figure 3D). The unstained colonies of SP and MP cells of SKOV3 from these plates were trypsinized and the cells were again plated in a six well tissue culture plate at 100 cells per well and grown for another 14 days and the CFE was estimated. SKOV3 MP cells had a CFE of 8.0±1.5% compared to a CFE of 44.0±4.0% for the SP cells. The original 2-fold difference in CFE between MP and SP of SKOV3 found to be increased to 5–fold after first passage (*P<0.001*) (Figure 3E). A similar trend was observed for A224 SP cells (Figure 3F). The increase in CFE as a function of passage indicates the enrichment of cancer stem-like cells present in the SP fraction of SKOV3 and A224, and its capability for sustained expansion.

**Figure 4:**
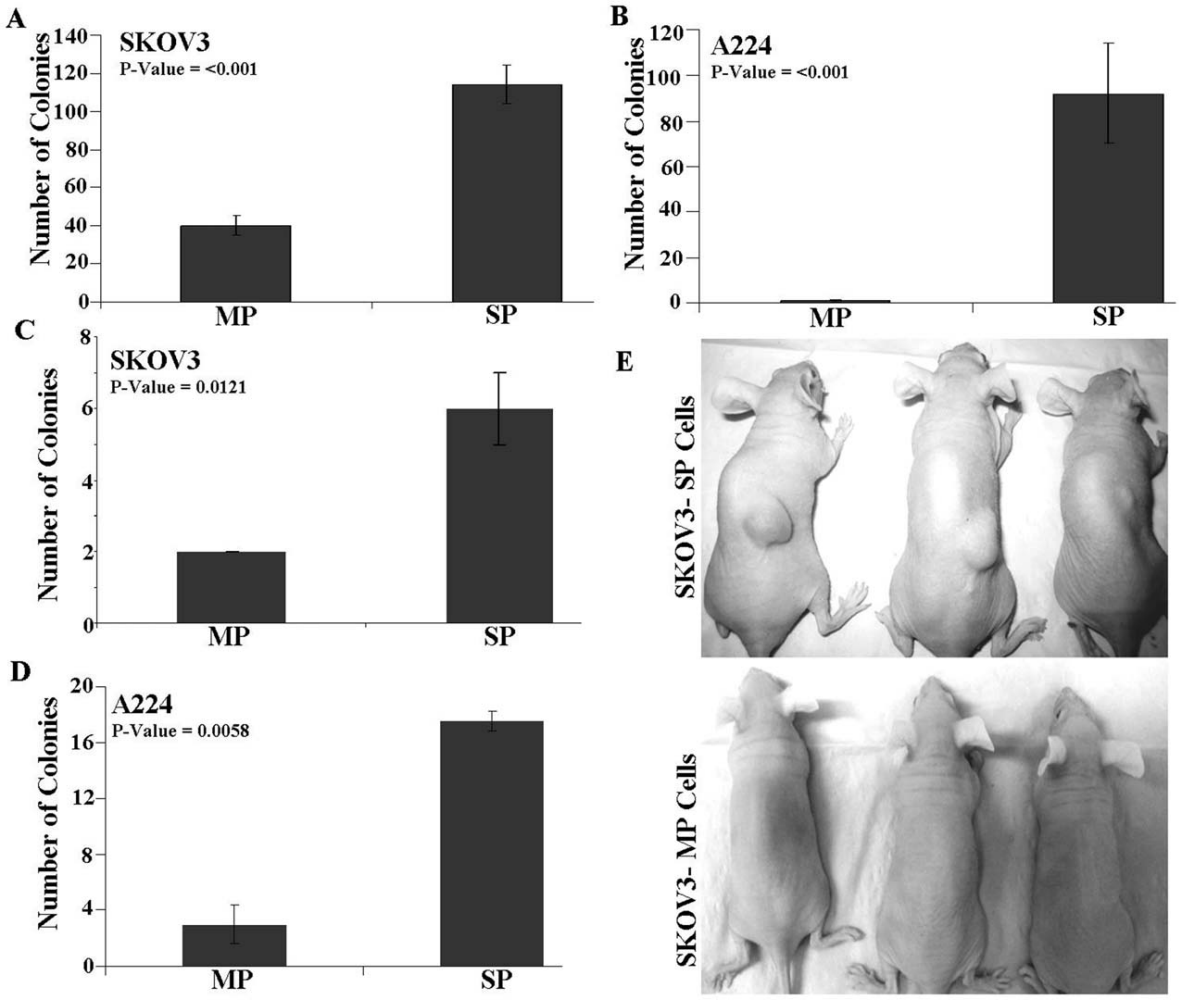
Biological validation of SP cells isolated from ovarian cancer cell lines. (A, B) Anchorage independent growth of the sorted SP and MP cells from SKOV3 and A224 cell lines. (C, D) Single cell assay in 96 well plates: Single cells of A224 and SKOV3 SP and MP cells were cultured in individual wells. The single cells were allowed to grow for 14 days and the colony forming efficiency was estimated using crystal violet staining. (E) *In vivo* tumor growth of SKOV3 SP and MP cells in female athymic nude mice.

To assess the anchorage independent growth of SP and MP cells from each cell lines, its ability to form colonies in soft agar were evaluated. The A224 SP cells formed approximately 90 colonies/5000 cells per dish, whereas A224 MP cells formed a couple of colonies only (*P<0.001*) yielding a plating efficiency of 1.8% to the SP cells. In case of SKOV3 cells, the plating efficiencies were 4.8% and 1.6% for SP and MP cells respectively (Figure 4A, B).

To ascertain single cell cloning efficiencies of SP and MP cells, single cell cultures of A224 and SKOV3 SP and MP cells were prepared using FACS into 96 well plates. The plates were allowed to grow for 14 days and the colony forming efficiency was estimated using crystal violet staining method. A224 MP cells had a CFE of 3.0±1.5% compared to a CFE of 17±1.0% for the A224 SP cells (*P = 0.005*), whereas SKOV3 MP cells had a CFE of 2.0% compared to a CFE of 6.0±1.0% for the SKOV3 SP cells (Figure 3C, D). In addition the colonies generated from SP cells were grown to more than 80% of each well while the colonies formed from the MP fractions were demonstrated limited growth in the wells (Figure S6).

**Figure 5:**
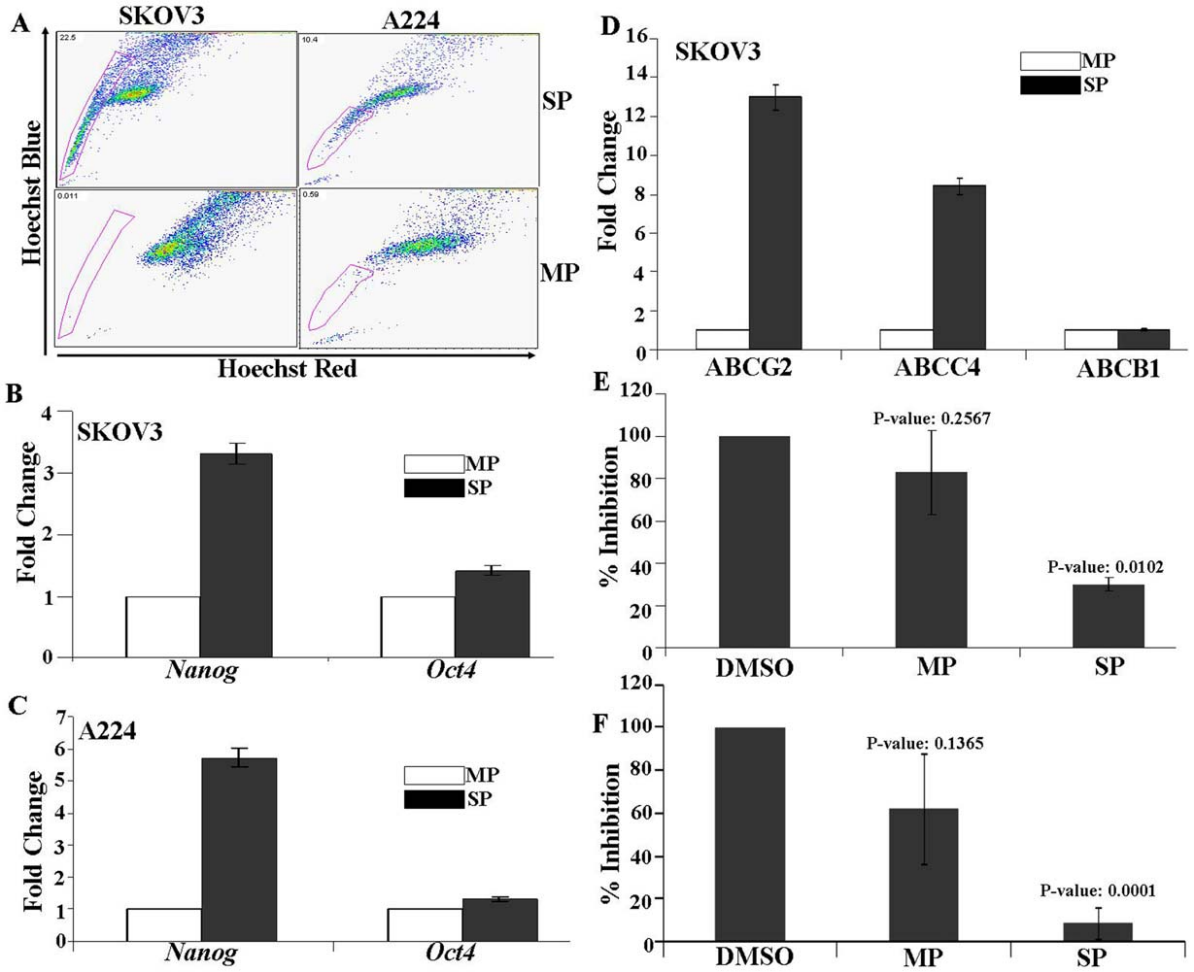
Validation of SP cells from cell lines and the effect of GSI-IX inhibitor on SP cells colony forming efficiency. (A) Repopulation Assay: The SKOV3 and A224 cells were stained with Hoechst 33342 dye and sorted for SP and MP populations. The cells were cultured for 8 days for repopulation, and then reanalyzed by flow cytometry. This demonstrated the enrichment of SP cells (22.5% and 10.4% for SKOV3 and A224 resp.) with a capacity to regenerate to MP cells. (B, C, D) qRT-PCR analysis for stem cell marker genes and transporter genes (p<0.01). To calculate the relative expression for each gene, the 2^−ΔΔCT^ method was used averaging the CT values for the three housekeeping genes (Cyclophilin A, GUSB, GAPDH) for a single reference gene value. (E, F) Inhibitory effect of GSI-IX on Colony forming efficiency of sorted SP and MP cells from SKOV3. Cells were sorted to SP and MP populations and treated with 10 and 20 µg of GSI-IX. The inhibitor carrier DMSO is used as a control.

To evaluate the *in vivo* tumorigenic potential of SP and MP cells from SKOV3, 5×10^4^ cells in 100 µL PBS were injected subcutaneously into the dorsal area of the nude mice. Tumor growth was noticed in three of three animals at 8 weeks after injection in SP cells, whereas animals injected with equal number of MP cells had no detectable tumors at that time (Figure 4E). To elucidate whether SP cells self renew and generate MP cells, sorted SP cells and MP cells were cultured separately under the same conditions for 8 days before being restained with Hoechst 33342 dye and reanalyzed. The results show that SP cells from both cell lines regenerated SP and MP, but the MP cells from both cell lines produced only MP cells (Figure 5A).

### Expression of stem cell marker genes, transporter genes and CD markers in SP and MP cells from the SKOV3 and A224 cell lines

Relative mRNA expression levels of stem cell markers (oct4 and nanog) and ABC transporter genes (ABCG2, ABCC4, ABCB1) were evaluated by qRT-PCR in the sorted SP and MP cells from SKOV3 and A224 cell lines. The expression of nanog, a gene associated with self-renewal, is elevated in both SKOV3 and A224 SP cells compared to the MP cells. A marginal upregulation was observed for oct4 in SKOV3 and A224 SP cells (*P<0.01*) (Figure 5B, C). The expression level of three different ABC transporters (ABCG2, ABCC4 and ABCB1) was evaluated. ABCG2 and ABCC4 were significantly up regulated in SKOV3 SP cells compared to its MP counterpart (Figure 5D). ABCG2 and ABCB1 were found to be upregulated in A224 SP cells (Figure S7). CD markers were evaluated using FACS. The relative expression of different cell surface markers on SKOV3 SP and MP cells is provided in table (Table S3). SP and MP cells found to strongly express tumor metastasis marker CD44. Compared to MP cells, the SKOV3 SP cells were showing strong expression of CD24 (gene encodes a sialoglycoprotein). SKOV3 MP cells showed higher expression of CD117/c-kit in compared to SP cells.

### Effect of γ-Secretase inhibitor GSI-IX on SKOV3 SP and MP cells

qRT-PCR using gene specific primers for γ-secretase (PSENEN) identified statistically significant overexpression of the gene in SP population in compared to its MP counterpart (Figure S8). The effect of γ-secretase inhibitors GSI-IX (inhibitor of Notch signaling pathway) on colony forming efficiency was evaluated on the sorted SKOV3 SP and MP cells. A 70% reduction of SP cells CFU efficiency (*P = 0.0102*) was observed at 10 µg of GSI-IX. Whereas at the same concentration a marginal reduction was observed for MP cells, which was found to be statistically insignificant (*P = 0.2567*) (Figure 5E). At a higher concentration of GSI-IX (20 µg) 92% inhibition was observed for SKOV3 SP (*P = 0.0001*) demonstrating the dose dependent sensitivity of SP cells to GSI-IX (Figure 5F).

### Enrichment of SP gene signature in recurrent ovarian cancer patients expression database

**Figure 6:**
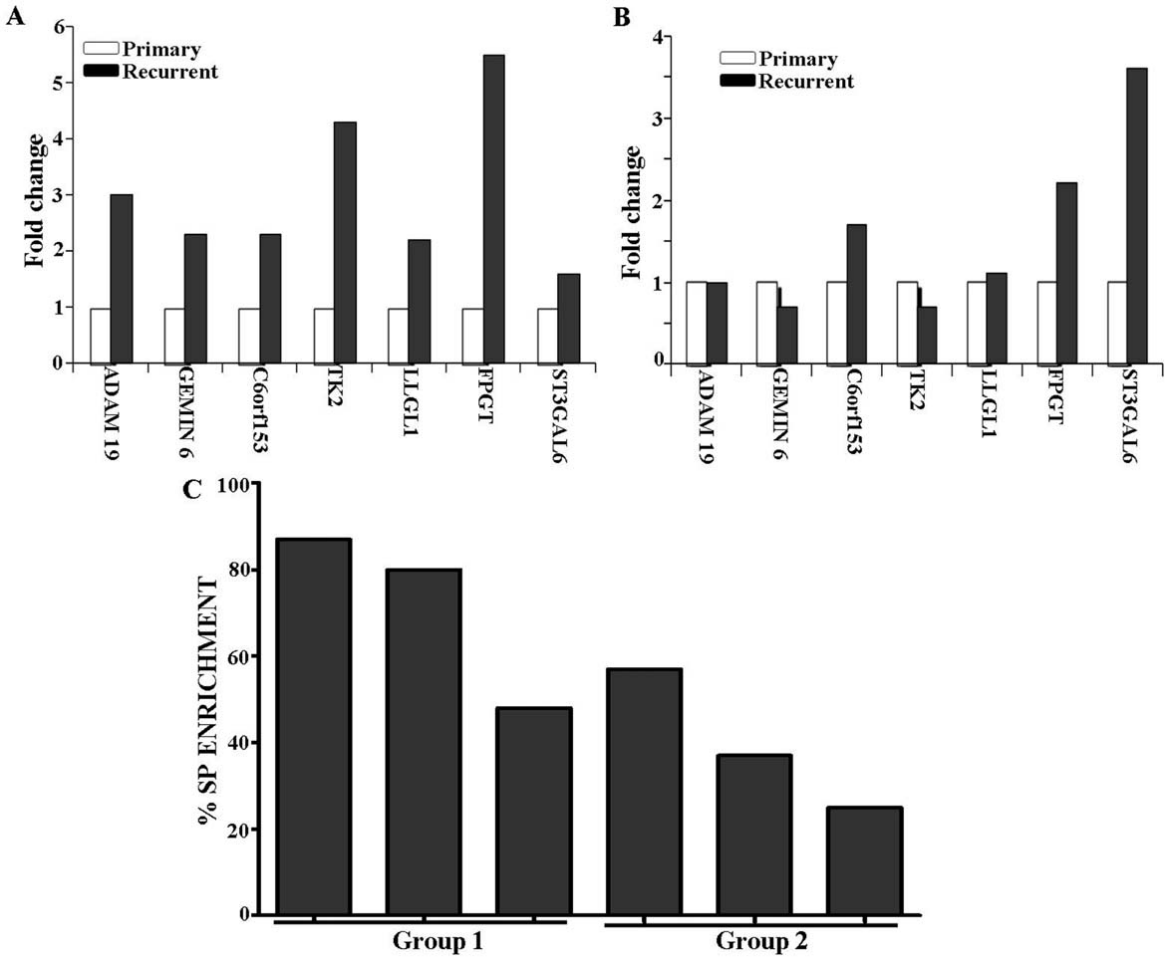
Validation of SP cell signature on ovarian tumor recurrent expression database. (A, B) qRT-PCR Validation of SP cell gene signature in recurrent ovarian cancer specimens. (A) Group 1 consists of tumor recurrence observed between 1 to 12 months and (B) group 2 in between 13 to 24 months. (C) Electronic validation of SP cell gene signature in recurrent ovarian cancer patients.

It is assumed that the stem cell (and their gene signature) would be enriched in recurrent ovarian cancer specimen. Six patient's expression files for 12 samples (primary and recurrent) and the respective mRNA were evaluated. The expression levels of randomly selected genes from SP gene list were evaluated on primary and recurrent ovarian tumor samples using qRT-PCR. All the genes evaluated were found to be over expressed in recurrent tumor compared to its primary counterpart. Further the tumor samples were categorized into two groups based on the time period of tumor recurrence. Group 1 consists of tumor recurrence observed between 1 to 12 months and group 2 in between 13 to 24 months. The qRT-PCR data shows the expression level of all the genes evaluated were high in group 1 compared to group 2 patients (Figure 6A, B). We found that 72% of SP cell gene signature enrichment for group 1 patients, and the same for group 2 patients were only 39%. Figure 6C shows the SP cell enrichment data for individual patients These results also further validate the enrichment of potential cancer stem-like cells in SP gene expression profile identified from ascites of patients with advanced ovarian cancer.

## Discussion

SP cells are a rare cell population that was initially used for the identification of hematopoietic stem cells and recently has been used to enrich for stem cells in a variety of tissue types [24], [43]. To specifically target SP cells, the knowledge of the basic biology of SP cells and the potential differences that exist between SP cells from the rest of the cell population must be elucidated. This study reports a SP cell gene expression signature from isolated SP of fresh ascites obtained from women with high-grade advanced stage papillary serous ovarian adenocarcinoma using Affymetrix U133 Plus 2.0 microarray platform. The array results identified 438 unique probe sets, which distinguished SP population from the remaining cancer cells (MP) (Table S4). Gene Ontology analysis on the array results revealed that the SP cell genes were associated with distinct biological processes including apoptosis, cell cycle, signal transduction, and cell proliferation which are different from the MP population. To validate the gene signature from the patient specimen and to support that the SP fraction contains stem-like cells we provide several lines of evidence. 1) Randomly selected genes from the patient SP gene list (ADAM19, FPGT, ST3GAL6, LLGL1 and TK2) were validated on SP cells isolated from cell lines. 2) Two stem cell related genes namely Nanog and Oct4 were found to be overexpressed in SP cells from cell lines. Nanog and Oct4 are reported to be transcriptional factors required to maintain the pluripotency and self-renewal of stem cells [44]. 3) The results of increased colony forming efficiency of SP cells isolated from SKOV3 and A224 cell lines compared with MP cells also confirmed the enrichment of potential cancer stem-like cells in SP cells. The CFE as a function of passage indicated the sustained expansion capacity of the SP population in compared to MP. The colony forming capacity of sorted single cells were in good correlation with the results of plate assays. 4) An increase in the anchorage independent growth of SP cells isolated from SKOV3 and A224 cell lines compared with MP cells were noticed. 5) Finally *in vivo* tumorigenic assays in nude mice suggest the SP cells have the potential to initiate the tumor growth at lower numbers.

Data mining for biologically relevant processes using Pathway Studio 6.0 identified overexpressed genes in SP cells that were related to functional cancer stem cell-like phenotypes. Further, the SP genes were enriched for genes associated with stem cell characteristics in different normal tissues. Genes implicated in normal stem cell function (NUP, ST3GAL, LTBP, KLF, TLE, NFAT, ATRX) were upregulated in SP. We found the over expression of EPHB4 and EPHB2 in SP gene list. EPHB4 is a transmembrane receptor tyrosine kinase and recently reported as a novel ovarian tumor marker and a viable target for biological therapy [45]. The overexpression of EPHB4 may be involved with the inhibition of CASP8 activation and subsequent CASP8 mediated apoptosis (Figure 2B). AKT1 was also found to be upregulated in SP cells and this gene may be involved in cellular survival pathways by inhibiting apoptosis process by a variety of routes [46], [47], [48], [49], [50]. Alterations in apoptotic pathways are one of the known mechanisms for the cancer stem cell survival [25].

Tissue stem cells use multiple signaling pathways to control normal stem cell self-renewal and deregulation of these pathways may produce neoplastic proliferation with the development of a cancer stem cell [51], [52]. The cleavage of the Notch transmembrane domain by ADAM proteases and presenilins are important for the activation of Notch pathway. ADAM proteases are considered to be multidomain proteins with multiple functions, involved in the proteolytic processing of other transmembrane proteins, cell adhesion and cell signaling events. The overexpression of ADAM8, ADAM9, ADAM10, ADAM12, ADAM15, ADAM17, and ADAM28 are reported from various cancers [53]. The extracellular domain of Notch receptors is glycosylated with N-glycans and O-glycans including fucose and glucose. The transfer of fucose is catalyzed by the enzyme fucosyltransferases (Pofut1), and the fucose may be extended with N-acetyl glucosamine (GlcNAc) and subsequently by galactose and sialic acid [54]. The important role of fucose on Notch signaling was first shown in Chinese Hamster Ovary (CHO) cells that make very low amount of GDP-fucose, a substrate for Pofut1 [55]. Hence the enzymes involved in the synthesis of GDP-fucose is also plays a key regulatory role in Notch signaling pathway. Fucose can be converted into GDP-Fucose through a salvage pathway by an enzyme namely fucose-1-phospate guanylyltransferase (FPGT). This GDP-Fucose can then serve as a substrate for Pofut1, which transfer fucose to Notch receptors and the subsequent activation of the Notch signaling pathway. ST3GAL6 (ST3 beta-galactoside alpha-2,3-sialyltransferase 6) catalyzes the transfer of sialic acid from cytidine 5-prime-monophosphate-N-acetyl neuraminic acid to terminal positions of glycoprotein and glycolipids. Chen et al reported that the cell surface glycans are essential for the cellular signal transduction in human hematopoietic stem and progenitor cells [41]. Based on these results and identification of these genes (FPGT, ST3GAL6 and ADAM19) in our SP gene list, we identified a potential mechanism, which may be active in the SP cells that accounts for the SP cell survival, differentiation and tumor maintenance in ovary (Figure 2C). The figure shows the involvement of three enzymes (FPGT, ST3GAL6 and ADAM19) in activation of Notch signaling pathway. The increased expression of these enzymes can lead to an increase in release of Notch intracellular domain translocated to the nuclease where it interacts with transcriptional complex and activates downstream target genes. We found that the Notch target genes such as *NOTCH1*, *HES1*, *NRARP* and *TCFL 5* were significantly upregulated in the SP fractions in compared to MP counterpart. These results indicate a difference in Notch activation and signaling between the SP and MP populations. These results indentify the notch pathway as a potential therapeutic target in ovarian cancer. The studies with specific γ-Secretase inhibitor (GSI-IX) demonstrate the mechanistic significance of Notch signaling pathway in the survival of ovarian SP cells and possible therapeutic intervention.

It has been reported that cancer stem cells contribute to chemoresistance and tumor progression in a variety of malignancies [5], [6]. If SP cells are enriched for stem-like cells then we would hypothesis that the SP gene signature would be prominent in tumor which recur quickly An enrichment of SP cell genes in recurrent ovarian tumor was identified in comparison to its primary tumor counterpart. The recurrence patient data was categorized into two groups based on the time period of tumor recurrence (Group 1 patient suffered recurrence between 1–12 months and Group 2 after 12 months). This grouping is associate with platinum sensitivity [56], [57]. The percentage of SP signature genes were detected more often tumors which recurred early versus late. This is associated with the concept that tumors which recur quickly contain more SP cells. SP cells isolated from SKOV3 and A224 cell lines demonstrated higher expression of different ABC transporter genes in comparison to its MP counter part. These transporter genes have been reported to provide protection to the cells from chemotoxic agents as well as from hypotoxic conditions [58], [59]. Of course Notch signaling as described above may be critical in tumor recurrence. Different cell surface markers are reported in associated to cancer stem cell properties in ovarian cancer [60], [61], [62], [63], [64], [65]. The heterogeneity in the phenotype of the cell surface markers between SP and MP populations were also evaluated.

In summary, an expression profile for SP enriched for cancer stem-like cells from ascites of ovarian cancer patients is reported. The nature of the “stemness” of the SP gene signature was demonstrated by the identification of several stem cell-related genes including an activated Notch signaling pathway. The results were biologically validated using identified SP population from human ovarian cancer cell lines. The SP gene list generated from ovarian cancer patients was also found to be enriched in recurrent tumors from ovarian cancer patients. These results have important implications concerning the tumor recurrence and potential therapeutic approach. The SP cells showed a dose dependent sensitivity towards Notch pathway inhibitor, suggests the Notch signaling pathway may be an important therapeutic target in ovarian cancer.

## Supporting Information

Figure S1 CA 125 staining for ascites samples: The CA 125 staining and analysis using FACS confirmed the ovarian origin of cells isolated from ascites and the ovarian cancer cell line SKOV3.
(DOC)

Figure S2 Representative graph for the SP and MP sort from ascites samples.
(DOC)

Figure S3 Pearson's and Spearman's analysis graph.
(DOC)

Figure S4 Western Blot Analysis of FPGT expression in SP and MP cells of SKOV3 and A224 cell lines (above). Immunoflourescence of ADAM19 in A224 SP and MP cells: The A224 cells were sorted for SP and MP and grown on the cover slip. The tissue culture media was removed and washed the wells with PBS twice. The cells were then fixed using 4% Para formaldehyde for 12 minutes and two quick washes were given with PBS. The cells were then permeabilized using 1% Triton in 0.02% BSA in PBS for 2 minutes. The cells were then incubated with blocking serum (20% heat inactivated serum with 2% BSA in PBS) for 20 minutes in room temperature. The cells were washed with PBS and control were incubated with Blocking Peptide (BP, ADAM19-P, SC-25989, Santa Cruz Biotechnology). The cells were stained with primary antibody (ADAM19 Goat plyclonal IgG, SC25989, Santa Cruz Biotechnology) for 2 hours at room temperature. The cells were washed thrice with PBS and stained for secondary antibody (Donkey anti-goat IgG-FITC, SC-2024, Santa Cruz Biotechnology) for 30 minutes at room temperature. The cells washed thrice with PBS and stained with DAPI for 5 minutes. SP and MP cells showed 64±4% and 33±5% positively stained cells for ADAM19. Representative figures showed above.
(DOC)

Figure S5 Expression level of Notch target genes in A224 and SKOV3 SP and MP cells.
(DOC)

Figure S6 Single cell colony forming assay.
(DOC)

Figure S7 Expression level of transporter genes in A224 SP cells.
(DOC)

Figure S8 Expression level of PSENEN in A224 and SKOV3 SP and MP cells.
(DOC)

Table S1 Pearson's and spearman's analysis results.
(DOC)

Table S2 Primer sequence information.
(DOC)

Table S3 The relative expression of different cell surface markers on SKOV3 SP and MP.
(DOC)

Table S4 SP gene list.
(DOC)
