# Supplementary figures and images for: Identification of a Potential Ovarian Cancer Stem Cell Gene Expression Profile from Advanced Stage Papillary Serous Ovarian Cancer
Source: PLoS One. 2012 Jan 17;7(1):e29079. doi: 10.1371/journal.pone.0029079 (PMC3260150; doi:10.1371/journal.pone.0029079)

**Figure S1:**


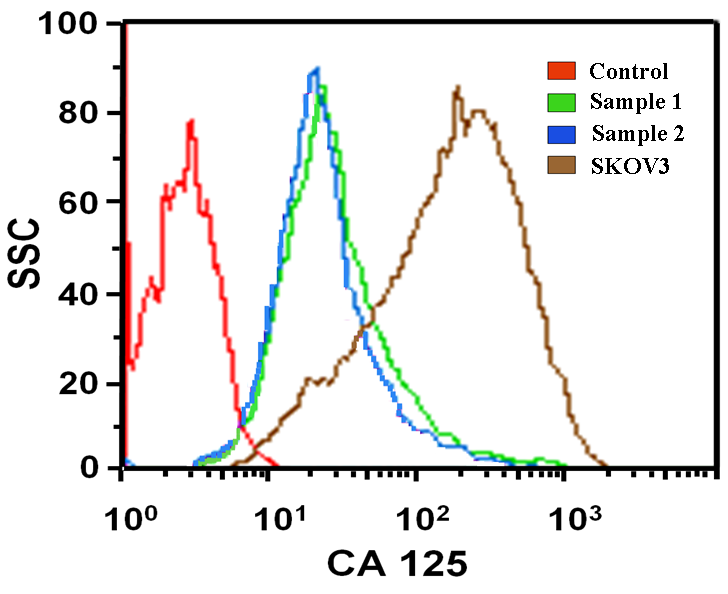

Supplement: Figure S1 — CA 125 staining for ascites samples: The CA 125 staining and analysis using FACS confirmed the ovarian origin of cells isolated from ascites and the ovarian cancer cell line SKOV3. (DOC) [file pone.0029079.s001.doc]

**Figure S3:**

**
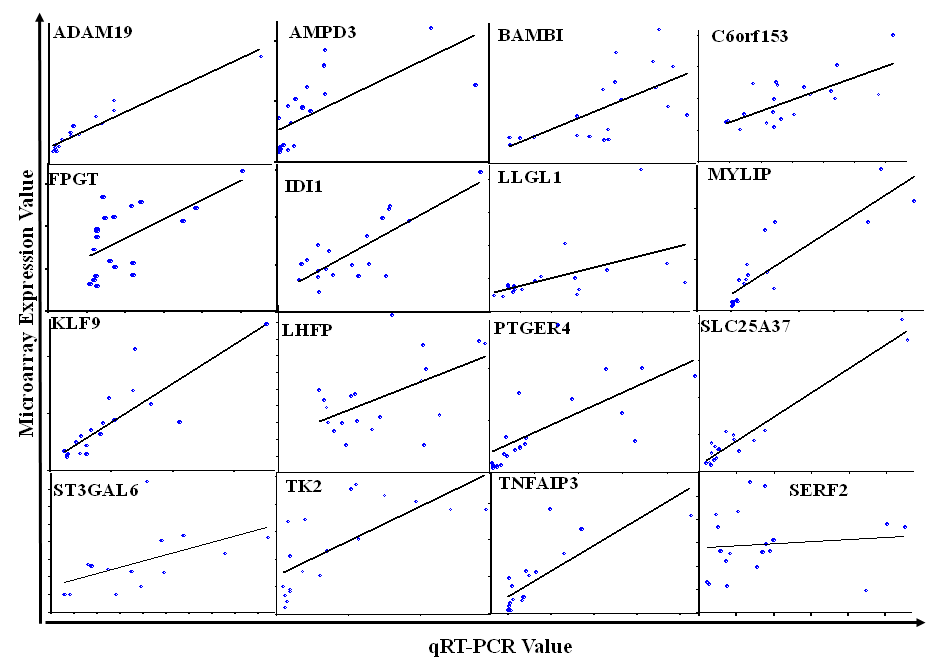
**

Supplement: Figure S3 — Pearson's and Spearman's analysis graph. (DOC) [file pone.0029079.s003.doc]

**
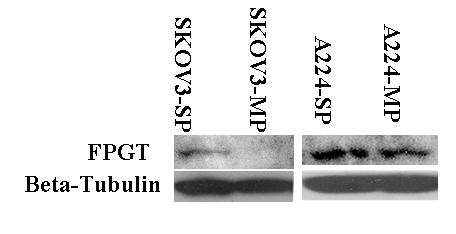
Figure S4:**


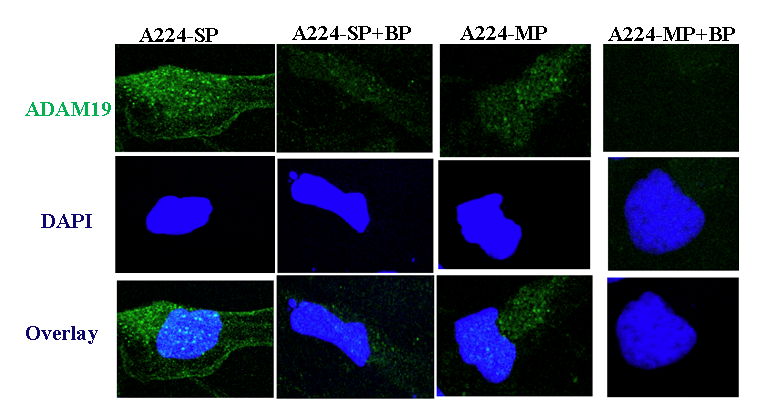

Supplement: Figure S4 — Western Blot Analysis of FPGT expression in SP and MP cells of SKOV3 and A224 cell lines (above). Immunoflourescence of ADAM19 in A224 SP and MP cells: The A224 cells were sorted for SP and MP and grown on the cover slip. The tissue culture media was removed and washed the wells with PBS twice. The cells were then fixed using 4% Para formaldehyde for 12 minutes and two quick washes were given with PBS. The cells were then permeabilized using 1% Triton in 0.02% BSA in PBS for 2 minutes. The cells were then incubated with blocking serum (20% heat inactivated serum with 2% BSA in PBS) for 20 minutes in room temperature. The cells were washed with PBS and control were incubated with Blocking Peptide (BP, ADAM19-P, SC-25989, Santa Cruz Biotechnology). The cells were stained with primary antibody (ADAM19 Goat plyclonal IgG, SC25989, Santa Cruz Biotechnology) for 2 hours at room temperature. The cells were washed thrice with PBS and stained for secondary antibody (Donkey anti-goat IgG-FITC, SC-2024, Santa Cruz Biotechnology) for 30 minutes at room temperature. The cells washed thrice with PBS and stained with DAPI for 5 minutes. SP and MP cells showed 64±4% and 33±5% positively stained cells for ADAM19. Representative figures showed above. (DOC) [file pone.0029079.s004.doc]

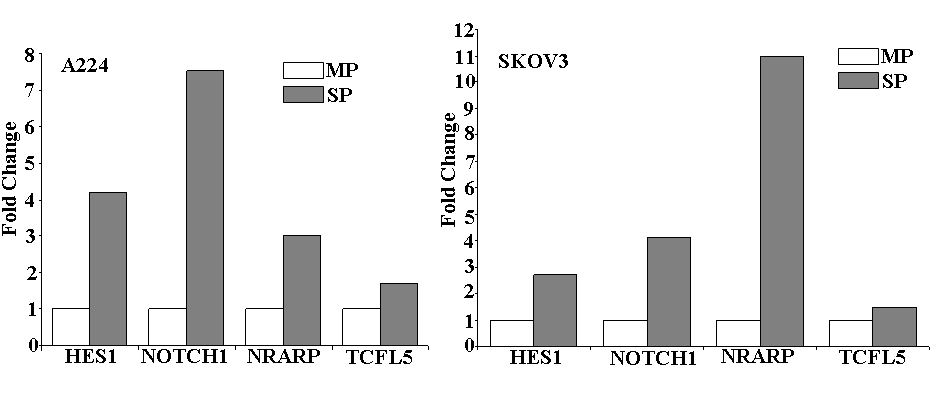
**Figure S5:**

Supplement: Figure S5 — Expression level of Notch target genes in A224 and SKOV3 SP and MP cells. (DOC) [file pone.0029079.s005.doc]

**Figure S6:**


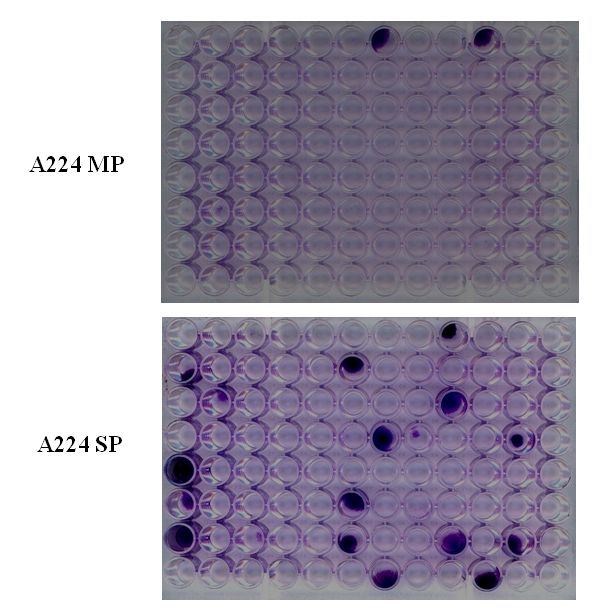

Supplement: Figure S6 — Single cell colony forming assay. (DOC) [file pone.0029079.s006.doc]

**Figure S8:**

**
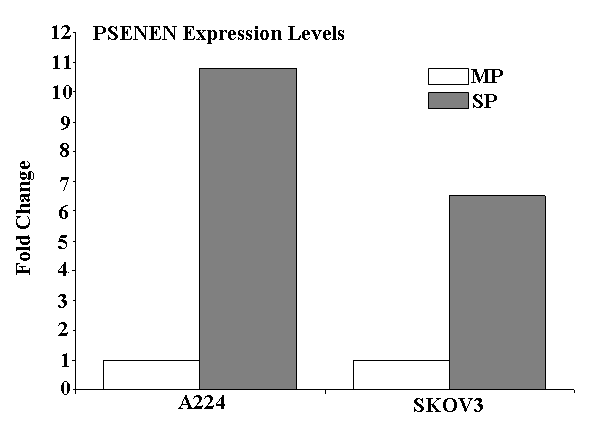
**

Supplement: Figure S8 — Expression level of PSENEN in A224 and SKOV3 SP and MP cells. (DOC) [file pone.0029079.s008.doc]
