# Supplementary material for: Identification of a Potential Ovarian Cancer Stem Cell Gene Expression Profile from Advanced Stage Papillary Serous Ovarian Cancer
Source: PLoS One. 2012 Jan 17;7(1):e29079. doi: 10.1371/journal.pone.0029079 (PMC3260150; doi:10.1371/journal.pone.0029079)
Supplement: Figure S2 — Representative graph for the SP and MP sort from ascites samples. (DOC) [file pone.0029079.s002.doc]

**Figure S2:**
