# Supplementary material for: Identification of a Potential Ovarian Cancer Stem Cell Gene Expression Profile from Advanced Stage Papillary Serous Ovarian Cancer
Source: PLoS One. 2012 Jan 17;7(1):e29079. doi: 10.1371/journal.pone.0029079 (PMC3260150; doi:10.1371/journal.pone.0029079)
Supplement: Figure S7 — Expression level of transporter genes in A224 SP cells. (DOC) [file pone.0029079.s007.doc]

**Figure S7:**

**MP**

**SP**
