# Supplementary material for: Identification of a Potential Ovarian Cancer Stem Cell Gene Expression Profile from Advanced Stage Papillary Serous Ovarian Cancer
Source: PLoS One. 2012 Jan 17;7(1):e29079. doi: 10.1371/journal.pone.0029079 (PMC3260150; doi:10.1371/journal.pone.0029079)
Supplement: Table S1 — Pearson's and spearman's analysis results. (DOC) [file pone.0029079.s009.doc]

**Table S1: Pearson’s and Spearman’s analysis results**

| **Gene** | **Array** | **qRT-PCR** | **Pearson’s Analysis** | | **Spearman’s Analysis** | |
| --- | --- | --- | --- | --- | --- | --- |
|  |  |  | **R value** | **P value** | **R value** | **P value** |
| *GEMIN6* | +1.90 | +2.57 | 0.8601 | <0.0001 | 0.8824 | <0.0001 |
| *KLF9* | -2.00 | -1.57 | 0.8455 | <0.0001 | 0.9030 | <0.0001 |
| *SLC25A37* | -3.33 | -2.70 | 0.9740 | <0.0001 | 0.8579 | <0.0001 |
| *MYLIP* | -3.33 | -1.50 | 0.8705 | <0.0001 | 0.9398 | <0.0001 |
| *PTGER4* | -2.50 | -1.51 | 0.6621 | 0.0015 | 0.8346 | <0.0001 |
| *IDI1* | -1.66 | 1.00 | 0.7936 | <0.0001 | 0.6130 | 0.0041 |
| *TK2* | +1.60 | +4.22 | 0.6948 | 0.0010 | 0.7925 | <0.0001 |
| *TNFAIP3* | -5.00 | -1.81 | 0.7882 | <0.0001 | 0.8737 | <0.0001 |
| *AMPD3* | -2.50 | -1.78 | 0.6744 | 0.0011 | 0.8605 | <0.0001 |
| *SERF2* | +2.10 | +1.84 | 0.1055 | 0.6672 | 0.2158 | 0.3749 |
| *GEM* | -3.33 | +2.70 | 0.2158 | 0.2180 | 0.3414 | 0.1408 |
| *C6orf153* | +1.70 | +1.53 | 0.7030 | 0.0005 | 0.6243 | 0.0033 |
| *FPGT* | +1.60 | +4.05 | 0.5985 | 0.0053 | 0.5609 | 0.0101 |
| *KITLG* | -2.00 | -1.74 | 0.7140 | 0.0004 | 0.7098 | 0.0005 |
| *LHFP* | -1.43 | -1.20 | 0.5387 | 0.0143 | 0.4090 | 0.0733 |
| *LLGL1* | +1.90 | +3.12 | 0.5126 | 0.0248 | 0.6053 | 0.0060 |
| *ST3GAL6* | +1.60 | +1.56 | 0.5090 | 0.0630 | 0.6336 | 0.0150 |
| *ADAM19* | +2.10 | +5.41 | 0.9611 | <0.0001 | 0.9388 | <0.0001 |
| *BAMBI* | -2.00 | -1.50 | 0.6469 | 0.0021 | 0.7068 | 0.0005 |
