# Supplementary material for: Identification of a Potential Ovarian Cancer Stem Cell Gene Expression Profile from Advanced Stage Papillary Serous Ovarian Cancer
Source: PLoS One. 2012 Jan 17;7(1):e29079. doi: 10.1371/journal.pone.0029079 (PMC3260150; doi:10.1371/journal.pone.0029079)
Supplement: Table S2 — Primer sequence information. (DOC) [file pone.0029079.s010.doc]

**Table S2: Primer Sequence**

| ***GENE*** | **Forward Primer (Seq 5’ to 3’)** | **Reverse Primer (Seq 5’ to 3’)** |
| --- | --- | --- |
| *PTGER4* | CCCAAACGTGGTTACATTAGCC | ACAGCATCACTGGGAGTTACAC |
| *AMPD3* | GGTGTTCTGTGAATCTCCTC | GCAGCCCCAGTGTAAAAC |
| *TK2* | GCTGGCATCCCTGTAGAG | TCTGCGGTGTACTGACTTC |
| *GEMIN6* | GCAAAGCATACAGCCCAGAG | CCCAGCCACACAGAGAGTC |
| *KLF9* | TGCGGAGCTTTGGGACACATC | GAAGGCTGGTTGCTGGGACTG |
| *IDI1* | GACATTTGGGCTGGATAAAACC | GGCATCTGGCAACATCCG |
| *SERF2* | GACAGAACGAGGGGACGTAA | TCGTTTGCCTTTTTCTGCTT |
| *LLGL1* | TGGGCTGGACAGTTGTTTC | CACACACACCTGACTAGGC |
| *C6orf153* | GCTAAGTCTTGGTCACATGGC | CCACATCATCTGAGCCCATTTC |
| *KITLG* | ATGGATGCGCAGATATACCC | AAAAGGGTTGGGACATACACC |
| *GEM* | GCAGGAAAGATCCCAAGTCAAACAG | GCCACACGCACCAGATCAAATAAC |
| *MYLIP* | GGTTGTCCCCTCTGTATGTTTC | CCGATAAGGTCATCTAGGTCTCC |
| *TNFAIP3* | AATGCCACAGTGTTCTCCTGAG | TGGGGAGGCAAGCAAAAGTG |
| *SLC25A37* | GCACCTTCCTCCAAAGTCCC | TGCTCACCCTTGAAACATACATTG |
| *ADAM19* | GGAGCCTGGATGGACAAGT | TCCAGGTCCAGGATCAGTTC |
| *LHFP* | GACATATGCATCCCCACACA | TTTGGCTTATTGGTCCATTT |
| *BAMBI* | GGATCAGCGGCAACAGATG | CACAGGTCAGACAGCAGTTC |
| *ST3GAL6* | GTCTATTGGGTGGCACCTGT | TCCCATAGGGCAAATCAAAC |
| *FPGT* | GGAAGTATCGCTGCGAGAAG | CAGGATCCACAAAAACGTGA |
| *ABCB1* | GAGGAAGACATGACCAGGT | GTCTTCCAGCTGCCAG |
| *ABCC4* | CATCCGAAGAATCCAGACCT | GGTCTCTGATGCCTTATCCC |
| *ABCG2* | CTTCGTTATTAGATGTCTTAGC | GTGCTCCATTTATCAGAACA |
| *Nanog* | AGTCCCAAAGGCAAACAACCCACTTC | ATCTGCTGGAGGCTGAGGTATTTCTGTCTC |
| *Oct4* | CATCAAAGCTCTGCAGAAAGAAC | CTGAATACCTTCCCAAATAGAACC |
| *GUSB* | ACTCTTGGTATCACGACTACG | CTGCTCCATACTCGCTCTTG |
| *GAPDH* | ACCCACTCCTCCACCTTTG | CACCACCCTGTTGCTGTAG |
| *Cyclophilin* | TTCTTCATCACCTATGGCAAAC | GCAACTTCTCCAACTCATCTAG |
