# Supplementary material for: Identification of a Potential Ovarian Cancer Stem Cell Gene Expression Profile from Advanced Stage Papillary Serous Ovarian Cancer
Source: PLoS One. 2012 Jan 17;7(1):e29079. doi: 10.1371/journal.pone.0029079 (PMC3260150; doi:10.1371/journal.pone.0029079)
Supplement: Table S3 — The relative expression of different cell surface markers on SKOV3 SP and MP. (DOC) [file pone.0029079.s011.doc]

**Table S3**

**The relative expression of different cell surface markers on SKOV3 SP and MP**

+

+

CD133

+++

+

CD117

+

+

CD34

++

++++

CD24

+++++

+++++

CD44

**MP (%)**

**SP (%)**

**Marker**

+ = <10%

++ = 10-30 %

+++ = 30-50%

++++ = 50-70%

+++++ = >70%
