# Supplementary material for: Identification of a Potential Ovarian Cancer Stem Cell Gene Expression Profile from Advanced Stage Papillary Serous Ovarian Cancer
Source: PLoS One. 2012 Jan 17;7(1):e29079. doi: 10.1371/journal.pone.0029079 (PMC3260150; doi:10.1371/journal.pone.0029079)
Supplement: Table S4 — SP gene list. (DOC) [file pone.0029079.s012.doc]

**Table S4: SP gene list**

| ***Gene symbol*** | **Probe set** | **P-Value** | **FDR** | **SP / MP Ratio** | **UG cluster** | **Map** | **Description** |
| --- | --- | --- | --- | --- | --- | --- | --- |
| *MYCT1* | 231947_at | 0.003883 | 0.357325 | 3 | Hs.18160 | 6q25.2 | Myc target 1 |
| *HIPK2* | 219028_at | 0.0013214 | 0.357325 | 2.3 | Hs.397465 | 7q32-q34 | homeodomain interacting protein kinase 2 (HIPK2), mRNA. |
| *ASB1* | 212819_at | 0.0077133 | 0.357325 | 2.2 | Hs.516788 | 2q37 | ankyrin repeat and SOCS box-containing 1 (ASB1), mRNA. |
|  | 228647_at | 0.0011451 | 0.357325 | 2.1 | Hs.655804 | 12 | Homo sapiens, clone IMAGE:3621928 |
| *SERF2* | 226692_at | 0.0024407 | 0.357325 | 2.1 | Hs.424126 | 15q15.3 | Small EDRK-rich factor 2 |
| *ADAM19* | 209765_at | 0.0074001 | 0.357325 | 2.1 | Hs.483944 | 5q32-q33 | ADAM metallopeptidase domain 19 (meltrin beta) (ADAM19), transcript variant 2, mRNA. |
| *FIGN* | 242828_at | 0.0025941 | 0.357325 | 2 | Hs.593650 | 2q24.3 | Fidgetin |
| *NQO1* | 201467_s_at | 0.003887 | 0.357325 | 2 | Hs.406515 | 16q22.1 | NAD(P)H dehydrogenase, quinone 1 (NQO1), transcript variant 1, mRNA. |
| *LOC728190* | 1558794_at | 0.0053547 | 0.357325 | 2 | Hs.655534 | 10q23.2 | Hypothetical protein LOC728190 |
| *GEMIN6* | 219539_at | 0.0005315 | 0.2865326 | 1.9 | Hs.143818 | 2p22.1 | gem (nuclear organelle) associated protein 6 (GEMIN6), mRNA. |
| *LLGL1* | 236594_at | 0.0027616 | 0.357325 | 1.9 | Hs.513983 | 17p11.2 | Lethal giant larvae homolog 1 (Drosophila) |
| *RGNEF* | 1554003_at | 0.0044277 | 0.357325 | 1.9 | Hs.482521 | 5q13.2 | Rho-guanine nucleotide exchange factor (RGNEF), mRNA. |
| *P18SRP* | 217608_at | 0.005323 | 0.357325 | 1.9 | Hs.656180 | 5q12.3 | P18SRP protein (P18SRP), mRNA. |
| *EIF5B* | 214313_s_at | 0.0000266 | 0.1504141 | 1.8 | Hs.158688 | 2q11.2 | eukaryotic translation initiation factor 5B (EIF5B), mRNA. |
| *C1orf43* | 208229_at | 0.0006959 | 0.305613 | 1.8 | Hs.287471 | 1q21.2 | chromosome 1 open reading frame 43 (C1orf43), transcript variant 2, mRNA. |
| *MRPL41* | 225425_s_at | 0.0013992 | 0.357325 | 1.8 | Hs.44017 | 9q34.3 | mitochondrial ribosomal protein L41 (MRPL41), nuclear gene encoding mitochondrial protein, mRNA. |
| *TNRC6B* | 1558142_at | 0.0034035 | 0.357325 | 1.8 | Hs.372082 | 22q13.1 | trinucleotide repeat containing 6B (TNRC6B), transcript variant 2, mRNA. |
| *ZNRD1* | 223639_s_at | 0.0054129 | 0.357325 | 1.8 | Hs.57813 | 6p21.3 | zinc ribbon domain containing 1 (ZNRD1), transcript variant b, mRNA. |
| *MAGEH1* | 218573_at | 0.005635 | 0.357325 | 1.8 | Hs.279819 | Xp11.21 | melanoma antigen family H, 1 (MAGEH1), mRNA. |
| *FLJ38984* | 212791_at | 0.007617 | 0.357325 | 1.8 | Hs.112023 | 1p34.3 | hypothetical protein FLJ38984 (FLJ38984), mRNA. |
| *KIAA1377* | 235956_at | 0.008312 | 0.357325 | 1.8 | Hs.156352 | 11q22.1 | KIAA1377 (KIAA1377), mRNA. |
| *TMEM17* | 1557137_at | 0.0002065 | 0.233361 | 1.7 | Hs.308028 | 2p15 | transmembrane protein 17 (TMEM17), mRNA. |
| *KRTHB5* | 226927_at | 0.0005206 | 0.2865326 | 1.7 | Hs.42547 | 12q23.3 | Keratin, hair, basic, 5 |
| *EPHB4* | 202894_at | 0.0016575 | 0.357325 | 1.7 | Hs.437008 | 7q22 | EPH receptor B4 (EPHB4), mRNA. |
| *SUHW2* | 230789_at | 0.0016714 | 0.357325 | 1.7 | Hs.43834 | 22q11.22 | suppressor of hairy wing homolog 2 (Drosophila) (SUHW2), mRNA. |
| *EPHB2* | 209589_s_at | 0.0023501 | 0.357325 | 1.7 | Hs.523329 | 1p36.1-p35 | EPH receptor B2 (EPHB2), transcript variant 1, mRNA. |
| *C6orf203* | 223576_at | 0.002729 | 0.357325 | 1.7 | Hs.486084 | 6q21 | chromosome 6 open reading frame 203 (C6orf203), mRNA. |
|  | 235938_at | 0.0033668 | 0.357325 | 1.7 | Hs.102276 | 2 | Transcribed locus |
| *LOC554203* | 1554448_at | 0.0034134 | 0.357325 | 1.7 | Hs.648327 | Xq13.2 | Hypothetical LOC554203 |
| *ACN9* | 218981_at | 0.0039821 | 0.357325 | 1.7 | Hs.592269 | 7q21.3 | ACN9 homolog (S. cerevisiae) (ACN9), mRNA. |
|  | 242273_at | 0.0039893 | 0.357325 | 1.7 | Hs.175569 | 12 | Transcribed locus |
| *NAPE-PLD* | 238722_x_at | 0.0047896 | 0.357325 | 1.7 | Hs.324271 | 7q22.1 | N-acyl-phosphatidylethanolamine-hydrolyzing phospholipase D (NAPE-PLD), mRNA. |
| *CSTA* | 204971_at | 0.0058276 | 0.357325 | 1.7 | Hs.518198 | 3q21 | cystatin A (stefin A) (CSTA), mRNA. |
| *LTBP1* | 202729_s_at | 0.0060426 | 0.357325 | 1.7 | Hs.654497 | 2p22-p21 | latent transforming growth factor beta binding protein 1 (LTBP1), transcript variant 2, mRNA. |
| *TCEAL8* | 224819_at | 0.0060969 | 0.357325 | 1.7 | Hs.389734 | Xq22.1 | transcription elongation factor A (SII)-like 8 (TCEAL8), transcript variant 2, mRNA. |
| *USP46* | 203869_at | 0.0061484 | 0.357325 | 1.7 | Hs.331478 | 4q12 | ubiquitin specific peptidase 46 (USP46), mRNA. |
| *NQO2* | 203814_s_at | 0.0062576 | 0.357325 | 1.7 | Hs.533050 | 6pter-q12 | NAD(P)H dehydrogenase, quinone 2 (NQO2), mRNA. |
| *DNM3* | 209839_at | 0.0069845 | 0.357325 | 1.7 | Hs.654775 | 1q24.3 | dynamin 3 (DNM3), mRNA. |
| *PHF23* | 223081_at | 0.0075666 | 0.357325 | 1.7 | Hs.647432 | 17p13.1 | PHD finger protein 23 (PHF23), mRNA. |
| *PIGC* | 202846_s_at | 0.0078002 | 0.357325 | 1.7 | Hs.188456 | 1q23-q25 | phosphatidylinositol glycan anchor biosynthesis, class C (PIGC), transcript variant 1, mRNA. |
| *YIPF5* | 224949_at | 0.0079177 | 0.357325 | 1.7 | Hs.372050 | 5q32 | Yip1 domain family, member 5 (YIPF5), transcript variant 2, mRNA. |
| *TP53I3* | 210609_s_at | 0.0085641 | 0.357325 | 1.7 | Hs.50649 | 2p23.3 | tumor protein p53 inducible protein 3 (TP53I3), transcript variant 2, mRNA. |
| *VANGL1* | 219330_at | 0.0096075 | 0.3735558 | 1.7 | Hs.515130 | 1p11-p13.1 | vang-like 1 (van gogh, Drosophila) (VANGL1), mRNA. |
| *RAB27A* | 235766_x_at | 0.0097427 | 0.3735558 | 1.7 | Hs.654978 | 15q15-q21.1 | RAB27A, member RAS oncogene family |
| *MSL3L1* | 207551_s_at | 0.0098016 | 0.3735558 | 1.7 | Hs.655288 | Xp22.3 | male-specific lethal 3-like 1 (Drosophila) (MSL3L1), transcript variant 2, mRNA. |
| *PCM1* | 228905_at | 0.0010711 | 0.357325 | 1.6 | Hs.491148 | 8p22-p21.3 | Pericentriolar material 1 |
| *ST3GAL6* | 213355_at | 0.0001571 | 0.2189306 | 1.6 | Hs.148716 | 3q12.1 | ST3 beta-galactoside alpha-2,3-sialytransferase 6 (ST3GAL6), mRNA |
| *FPGT* | 205140_at | 0.0023576 | 0.357325 | 1.6 | Hs.632389 | 1p31.1 | fucose-1-phosphate guanylyltransferase (FPGT), mRNA. |
| *CCDC131* | 213065_at | 0.0023875 | 0.357325 | 1.6 | Hs.527874 | 12q21.1 | coiled-coil domain containing 131 (CCDC131), mRNA. |
| *DOK1* | 216835_s_at | 0.0026437 | 0.357325 | 1.6 | Hs.103854 | 2p13 | docking protein 1, 62kDa (downstream of tyrosine kinase 1) (DOK1), mRNA. |
| *EPM2AIP1* | 227847_at | 0.0027566 | 0.357325 | 1.6 | Hs.28020 | 3p22.1 | EPM2A (laforin) interacting protein 1 (EPM2AIP1), mRNA. |
| *TK2* | 204276_at | 0.0047747 | 0.357325 | 1.6 | Hs.512619 | 16q22-q23.1 | thymidine kinase 2, mitochondrial (TK2), mRNA. |
| *TNFRSF1A* | 207643_s_at | 0.0049834 | 0.357325 | 1.6 | Hs.279594 | 12p13.2 | tumor necrosis factor receptor superfamily, member 1A (TNFRSF1A), mRNA. |
| *B3GALT6* | 225733_at | 0.0050574 | 0.357325 | 1.6 | Hs.284284 | 1p36.33 | UDP-Gal:betaGal beta 1,3-galactosyltransferase polypeptide 6 (B3GALT6), mRNA. |
| *CROT* | 231102_at | 0.0050783 | 0.357325 | 1.6 | Hs.125039 | 7q21.1 | Carnitine O-octanoyltransferase |
|  | 233369_at | 0.0053122 | 0.357325 | 1.6 | Hs.659112 | 14 | CDNA FLJ11835 fis, clone HEMBA1006595 |
| *HCG12* | 227980_at | 0.0053674 | 0.357325 | 1.6 |  | 6p21.3 | HLA complex group 12 (HCG12) on chromosome 6. |
| *MTERFD2* | 1557965_at | 0.0057467 | 0.357325 | 1.6 | Hs.159556 | 2q37.3 | MTERF domain containing 2 |
| *PRPF38A* | 223230_at | 0.0069595 | 0.357325 | 1.6 | Hs.5301 | 1p33-p32.1 | PRP38 pre-mRNA processing factor 38 (yeast) domain containing A (PRPF38A), transcript variant 2, mRNA. |
| *BRI3BP* | 231810_at | 0.0076539 | 0.357325 | 1.6 | Hs.632740 | 12q24.31 | BRI3 binding protein |
| *ATF7* | 228829_at | 0.0078128 | 0.357325 | 1.6 | Hs.12286 | 12q13 | Activating transcription factor 7 |
| *FOXN3* | 230790_x_at | 0.0079681 | 0.357325 | 1.6 |  | 14q31.3 | forkhead box N3 (FOXN3), transcript variant 1, mRNA. |
| *SNRPF* | 203832_at | 0.0080202 | 0.357325 | 1.6 | Hs.105465 | 12q22 | small nuclear ribonucleoprotein polypeptide F (SNRPF), mRNA. |
| *P18SRP* | 227288_at | 0.0086766 | 0.3587496 | 1.6 | Hs.656180 | 5q12.3 | P18SRP protein (P18SRP), mRNA. |
| *C6orf153* | 235223_at | 0.0092776 | 0.3688657 | 1.6 | Hs.309231 | 6p21.1 | Chromosome 6 open reading frame 153 |
| *POLI* | 238992_at | 0.0098077 | 0.3735558 | 1.6 | Hs.438533 | 18q21.1 | Polymerase (DNA directed) iota |
| *SLC35D2* | 213083_at | 0.0016697 | 0.357325 | 1.5 | Hs.654897 | 9q22.32 | solute carrier family 35, member D2 (SLC35D2), mRNA. |
| *GOLGA2* | 204384_at | 0.0022635 | 0.357325 | 1.5 | Hs.155827 | 9q34.11 | golgi autoantigen, golgin subfamily a, 2 (GOLGA2), mRNA. |
| *NUP50* | 218294_s_at | 0.0025784 | 0.357325 | 1.5 | Hs.475103 | 22q13.31 | nucleoporin 50kDa (NUP50), transcript variant 3, mRNA. |
| *EPHB2* | 210651_s_at | 0.0025935 | 0.357325 | 1.5 | Hs.523329 | 1p36.1-p35 | EPH receptor B2 (EPHB2), transcript variant 1, mRNA. |
| *KIAA2013* | 224708_at | 0.002807 | 0.357325 | 1.5 | Hs.520094 | 1p36.22 | KIAA2013 |
| *STOML2* | 215416_s_at | 0.002934 | 0.357325 | 1.5 | Hs.3439 | 9p13.1 | stomatin (EPB72)-like 2 (STOML2), mRNA. |
| *SLC25A20* | 203658_at | 0.0031346 | 0.357325 | 1.5 | Hs.13845 | 3p21.31 | solute carrier family 25 (carnitine/acylcarnitine translocase), member 20 (SLC25A20), nuclear gene encoding mitochondrial protein, mRNA. |
| *FLJ10038* | 205510_s_at | 0.0032578 | 0.357325 | 1.5 | Hs.511316 | 15q15.3 | Hypothetical protein FLJ10038 |
| *MORC2* | 216863_s_at | 0.0033636 | 0.357325 | 1.5 | Hs.555918 | 22q12.2 | MORC family CW-type zinc finger 2 (MORC2), mRNA. |
| *HTATIP2* | 210253_at | 0.0039718 | 0.357325 | 1.5 | Hs.90753 | 11p15.1 | HIV-1 Tat interactive protein 2, 30kDa |
| *C6orf89* | 225729_at | 0.0040706 | 0.357325 | 1.5 | Hs.433381 | 6p21.2 | chromosome 6 open reading frame 89 (C6orf89), mRNA. |
| *MRPL14* | 225201_s_at | 0.0042225 | 0.357325 | 1.5 | Hs.311190 | 6p21.3 | mitochondrial ribosomal protein L14 (MRPL14), nuclear gene encoding mitochondrial protein, mRNA. |
| *CBR1* | 209213_at | 0.004874 | 0.357325 | 1.5 | Hs.88778 | 21q22.13 | carbonyl reductase 1 (CBR1), mRNA. |
| *RBM15B* | 226987_at | 0.0050961 | 0.357325 | 1.5 | Hs.476291 | 3p21.2 | RNA binding motif protein 15B (RBM15B), mRNA. |
| *MRPL16* | 217980_s_at | 0.0051361 | 0.357325 | 1.5 | Hs.530734 | 11q12-q13.1 | mitochondrial ribosomal protein L16 (MRPL16), nuclear gene encoding mitochondrial protein, mRNA. |
| *PTAR1* | 235484_at | 0.0059159 | 0.357325 | 1.5 | Hs.494100 | 9q21.11 | Protein prenyltransferase alpha subunit repeat containing 1 |
| *DLG3* | 212729_at | 0.0064187 | 0.357325 | 1.5 | Hs.522680 | Xq13.1 | discs, large homolog 3 (neuroendocrine-dlg, Drosophila) (DLG3), transcript variant 2, mRNA. |
| *TMEM57* | 241364_at | 0.006544 | 0.357325 | 1.5 | Hs.189782 | 1p36.11|1p36.11 | Transmembrane protein 57 |
| *ALG14* | 1553954_at | 0.0067852 | 0.357325 | 1.5 | Hs.408927 | 1p21.3 | asparagine-linked glycosylation 14 homolog (S. cerevisiae) (ALG14), mRNA. |
| *VPS4A* | 217913_at | 0.0075772 | 0.357325 | 1.5 | Hs.128420 | 16q22.1 | vacuolar protein sorting 4 homolog A (S. cerevisiae) (VPS4A), mRNA. |
| *AGGF1* | 208042_at | 0.0082254 | 0.357325 | 1.5 | Hs.634849 | 5q13.3 | angiogenic factor with G patch and FHA domains 1 (AGGF1), mRNA. |
| *CCM2* | 223164_at | 0.0085196 | 0.357325 | 1.5 | Hs.148272 | 7p13 | cerebral cavernous malformation 2 (CCM2), transcript variant 2, mRNA. |
| *SNX19* | 202359_s_at | 0.008734 | 0.3587496 | 1.5 | Hs.444024 | 11q25 | sorting nexin 19 (SNX19), mRNA. |
|  | 222518_at | 0.0000109 | 0.1504141 | 1.4 | Hs.649021 | 20 | Transcribed locus |
| *MRPS16* | 222499_at | 0.0012027 | 0.357325 | 1.4 | Hs.180312 | 10q22.1 | mitochondrial ribosomal protein S16 (MRPS16), nuclear gene encoding mitochondrial protein, mRNA. |
| *C10orf35* | 226313_at | 0.0012993 | 0.357325 | 1.4 | Hs.522992 | 10q21.3 | chromosome 10 open reading frame 35 (C10orf35), mRNA. |
|  | 1558517_s_at | 0.001761 | 0.357325 | 1.4 | Hs.412836 | 1 | CDNA FLJ37485 fis, clone BRAWH2014379 |
| *PFDN4* | 205362_s_at | 0.0021093 | 0.357325 | 1.4 | Hs.91161 | 20q13.2 | prefoldin subunit 4 (PFDN4), mRNA. |
| *TMEM182* | 238867_at | 0.0027338 | 0.357325 | 1.4 | Hs.436203 | 2q12.1 | transmembrane protein 182 (TMEM182), mRNA. |
| *TRAM2* | 202368_s_at | 0.0031899 | 0.357325 | 1.4 | Hs.520182 | 6p21.1-p12 | translocation associated membrane protein 2 (TRAM2), mRNA. |
| *LTV1* | 225748_at | 0.0032649 | 0.357325 | 1.4 | Hs.185675 | 6q24.2 | LTV1 homolog (S. cerevisiae) (LTV1), mRNA. |
| *LOC116143* | 235071_at | 0.0036588 | 0.357325 | 1.4 | Hs.631877 | 2p14 | monad (LOC116143), mRNA. |
|  | 229544_at | 0.0046211 | 0.357325 | 1.4 | Hs.167087 | 3 | CDNA clone IMAGE:4791887 |
| *FKBP11* | 219118_at | 0.005504 | 0.357325 | 1.4 | Hs.655103 | 12q13.12 | FK506 binding protein 11, 19 kDa (FKBP11), mRNA. |
| *BRCC3* | 221196_x_at | 0.0056518 | 0.357325 | 1.4 | Hs.558537 | Xq28 | BRCA1/BRCA2-containing complex, subunit 3 (BRCC3), transcript variant 2, mRNA. |
| *C18orf37* | 229582_at | 0.0060582 | 0.357325 | 1.4 | Hs.464903 | 18q12.2 | chromosome 18 open reading frame 37 (C18orf37), mRNA. |
| *DHX32* | 218198_at | 0.0063066 | 0.357325 | 1.4 | Hs.501379 | 10q26.2 | DEAH (Asp-Glu-Ala-His) box polypeptide 32 (DHX32), mRNA. |
|  | 226230_at | 0.0063431 | 0.357325 | 1.4 | Hs.654969 | 2 | Transcribed locus |
| *SEC11C* | 223299_at | 0.0065098 | 0.357325 | 1.4 | Hs.45107 | 18q21.32 | SEC11 homolog C (S. cerevisiae) (SEC11C), mRNA. |
| *SEPHS1* | 208941_s_at | 0.0065494 | 0.357325 | 1.4 | Hs.124027 | 10p14 | selenophosphate synthetase 1 (SEPHS1), mRNA. |
| *BTF3L4* | 226963_at | 0.00718 | 0.357325 | 1.4 | Hs.538093 | 1p32.3 | basic transcription factor 3-like 4 (BTF3L4), mRNA. |
| *GULP1* | 204237_at | 0.0073445 | 0.357325 | 1.4 | Hs.470887 | 2q32.3-q33 | GULP, engulfment adaptor PTB domain containing 1 (GULP1), mRNA. |
| *CDK5* | 204247_s_at | 0.0073697 | 0.357325 | 1.4 | Hs.647078 | 7q36 | cyclin-dependent kinase 5 (CDK5), mRNA. |
| *FANCF* | 218689_at | 0.0078844 | 0.357325 | 1.4 | Hs.632151 | 11p15 | Fanconi anemia, complementation group F (FANCF), mRNA. |
| *R3HCC1* | 35156_at | 0.0079721 | 0.357325 | 1.4 | Hs.458644 | 8p21.3 | R3H domain and coiled-coil containing 1 |
| *HEBP2* | 203430_at | 0.0082308 | 0.357325 | 1.4 | Hs.486589 | 6q24 | heme binding protein 2 (HEBP2), mRNA. |
| *SSR3* | 217790_s_at | 0.0087615 | 0.3590099 | 1.4 | Hs.518346 | 3q25.31 | signal sequence receptor, gamma (translocon-associated protein gamma) (SSR3), mRNA. |
| *COX18* | 227442_at | 0.0090962 | 0.3669665 | 1.4 | Hs.356697 | 4q13.3 | COX18 cytochrome c oxidase assembly homolog (S. cerevisiae) (COX18), mRNA. |
| *GGCX* | 235413_at | 0.009429 | 0.3728521 | 1.4 | Hs.77719 | 2p12 | gamma-glutamyl carboxylase (GGCX), mRNA. |
| *ATRX* | 208860_s_at | 0.0097503 | 0.3735558 | 1.4 | Hs.533526 | Xq13.1-q21.1 | alpha thalassemia/mental retardation syndrome X-linked (RAD54 homolog, S. cerevisiae) (ATRX), transcript variant 2, mRNA. |
| *PINX1* | 223907_s_at | 0.0000921 | 0.2220164 | 1.3 | Hs.490991 | 8p23 | PIN2-interacting protein 1 (PINX1), mRNA. |
| *KRT10* | 207023_x_at | 0.0001632 | 0.233361 | 1.3 | Hs.99936 | 17q21 | keratin 10 (epidermolytic hyperkeratosis; keratosis palmaris et plantaris) (KRT10), mRNA. |
| *UBE3B* | 213822_s_at | 0.0004424 | 0.2680312 | 1.3 | Hs.374067 | 12q24.11 | Ubiquitin protein ligase E3B |
| *MRPL43* | 224332_s_at | 0.0008724 | 0.3148807 | 1.3 | Hs.421848 | 10q24.31 | mitochondrial ribosomal protein L43 (MRPL43), nuclear gene encoding mitochondrial protein, transcript variant 1, mRNA. |
|  | 216384_x_at | 0.0018442 | 0.357325 | 1.3 | Hs.182314 | 12 | CDNA FLJ44260 fis, clone TLIVE2000979 |
| *PNPO* | 218511_s_at | 0.0026347 | 0.357325 | 1.3 | Hs.631742 | 17q21.32 | pyridoxamine 5'-phosphate oxidase (PNPO), mRNA. |
| *E2F3* | 203692_s_at | 0.0041725 | 0.357325 | 1.3 | Hs.269408 | 6p22 | E2F transcription factor 3 (E2F3), mRNA. |
| *C9orf105* | 228053_s_at | 0.0047915 | 0.357325 | 1.3 | Hs.130774 | 9 | PREDICTED: chromosome 9 open reading frame 105, transcript variant 6 (C9orf105), mRNA. |
| *CHMP4C* | 226803_at | 0.004805 | 0.357325 | 1.3 | Hs.183861 | 8q21.13 | chromatin modifying protein 4C (CHMP4C), mRNA. |
|  | 213158_at | 0.005837 | 0.357325 | 1.3 | Hs.592414 | 3 | Homo sapiens, clone IMAGE:4214654, mRNA |
| *CALM3* | 1563431_x_at | 0.005945 | 0.357325 | 1.3 | Hs.515487 | 19q13.2-q13.3 | calmodulin 3 (phosphorylase kinase, delta) (CALM3), mRNA. |
| *PFN2* | 204992_s_at | 0.0062311 | 0.357325 | 1.3 | Hs.91747 | 3q25.1-q25.2 | profilin 2 (PFN2), transcript variant 2, mRNA. |
| *DENR* | 234915_s_at | 0.0065922 | 0.357325 | 1.3 | Hs.22393 | 12q24.31 | density-regulated protein (DENR), mRNA. |
| *ADCK2* | 44120_at | 0.0069271 | 0.357325 | 1.3 | Hs.534141 | 7q32-q34 | aarF domain containing kinase 2 (ADCK2), mRNA. |
|  | 237419_at | 0.0072298 | 0.357325 | 1.3 | Hs.61481 | 8 | Transcribed locus |
| *PAWR* | 204005_s_at | 0.0072369 | 0.357325 | 1.3 | Hs.643130 | 12q21 | PRKC, apoptosis, WT1, regulator (PAWR), mRNA. |
| *GPR177* | 228950_s_at | 0.0073609 | 0.357325 | 1.3 | Hs.647659 | 1p31.3 | G protein-coupled receptor 177 (GPR177), transcript variant 2, mRNA. |
|  | 233946_at | 0.0074019 | 0.357325 | 1.3 | Hs.679417 | 9 | MRNA; cDNA DKFZp761L0916 (from clone DKFZp761L0916) |
| *SNRPE* | 215450_at | 0.0081718 | 0.357325 | 1.3 | Hs.334612 | 1q32 | small nuclear ribonucleoprotein polypeptide E (SNRPE), mRNA. |
| *CSNK1A1* | 243338_at | 0.0088855 | 0.3632135 | 1.3 | Hs.529862 | 5q32 | Casein kinase 1, alpha 1 |
| *SIPA1L3* | 213600_at | 0.009346 | 0.3704335 | 1.3 | Hs.655502 | 19q13.13 | signal-induced proliferation-associated 1 like 3 (SIPA1L3), mRNA. |
|  | 222883_at | 0.0096929 | 0.3735558 | 1.3 | Hs.349905 | 1 | Transcribed locus |
| *RPL35A* | 215208_x_at | 0.000177 | 0.233361 | 1.2 | Hs.529631 | 3q29-qter | Ribosomal protein L35a |
| *COMMD6* | 225312_at | 0.0047906 | 0.357325 | 1.2 | Hs.508266 | 13q22 | COMM domain containing 6 (COMMD6), transcript variant 2, mRNA. |
| *RPL35A* | 225190_x_at | 0.007071 | 0.357325 | 1.1 | Hs.529631 | 3q29-qter | ribosomal protein L35a (RPL35A), mRNA. |
| *TMEM1* | 208184_s_at | 0.008326 | 0.357325 | 1.1 | Hs.126221 | 21q22.3 | transmembrane protein 1 (TMEM1), transcript variant 1, mRNA. |
| *TJAP1* | 47608_at | 0.0000371 | 0.1573411 | -1.25 | Hs.520145 | 6p21.1 | tight junction associated protein 1 (peripheral) (TJAP1), mRNA. |
| *CEP76* | 52285_f_at | 0.0001047 | 0.2220164 | -1.25 | Hs.236940 | 18p11.21 | centrosomal protein 76kDa (CEP76), mRNA. |
| *CRTAP* | 1555889_a_at | 0.0005405 | 0.2865326 | -1.25 | Hs.517888 | 3p22.3 | cartilage associated protein (CRTAP), mRNA. |
| *MLLT4* | 224685_at | 0.0009849 | 0.3409764 | -1.25 | Hs.644024 | 6q27 | myeloid/lymphoid or mixed-lineage leukemia (trithorax homolog, Drosophila); translocated to, 4 (MLLT4), transcript variant 1, mRNA. |
| *TUFT1* | 205807_s_at | 0.0020881 | 0.357325 | -1.25 | Hs.489922 | 1q21 | tuftelin 1 (TUFT1), mRNA. |
| *CPD* | 201940_at | 0.0026 | 0.357325 | -1.25 | Hs.446079 | 17q11.2 | carboxypeptidase D (CPD), mRNA. |
| *RAB31* | 217764_s_at | 0.0028453 | 0.357325 | -1.25 | Hs.99528 | 18p11.3 | RAB31, member RAS oncogene family (RAB31), mRNA. |
| *KIAA0907* | 202220_at | 0.0030134 | 0.357325 | -1.25 | Hs.24656 | 1q22 | KIAA0907 (KIAA0907), mRNA. |
| *PTPN1* | 202716_at | 0.0034364 | 0.357325 | -1.25 | Hs.417549 | 20q13.1-q13.2 | protein tyrosine phosphatase, non-receptor type 1 (PTPN1), mRNA. |
| *HSPC268* | 226780_s_at | 0.0036682 | 0.357325 | -1.25 | Hs.370475 | 7q34 | hypothetical protein HSPC268 (HSPC268), mRNA. |
| *KIAA0355* | 203288_at | 0.0045965 | 0.357325 | -1.25 | Hs.330073 | 19q13.11 | KIAA0355 (KIAA0355), mRNA. |
| *C21orf66* | 218515_at | 0.0052004 | 0.357325 | -1.25 | Hs.693644 | 21q21.3 | chromosome 21 open reading frame 66 (C21orf66), transcript variant 1, mRNA. |
| *TNPO1* | 207657_x_at | 0.0054918 | 0.357325 | -1.25 | Hs.482497 | 5q13.2 | transportin 1 (TNPO1), transcript variant 2, mRNA. |
| *GNAI2* | 201040_at | 0.0058085 | 0.357325 | -1.25 | Hs.77269 | 3p21 | guanine nucleotide binding protein (G protein), alpha inhibiting activity polypeptide 2 (GNAI2), mRNA. |
| *ZNF138* | 244743_x_at | 0.0062683 | 0.357325 | -1.25 | Hs.184080 | 7q11.21-q11.23 | zinc finger protein 138 (ZNF138), mRNA. |
|  | 1558710_at | 0.0072578 | 0.357325 | -1.25 | Hs.655894 | 15 | CDNA FLJ40669 fis, clone THYMU2020883 |
|  | 227187_at | 0.007582 | 0.357325 | -1.25 | Hs.594184 | 7 | Full-length cDNA clone CS0CAP007YE04 of Thymus of Homo sapiens (human) |
| *DDEF2* | 206414_s_at | 0.0080436 | 0.357325 | -1.25 | Hs.555902 | 2p25 | development and differentiation enhancing factor 2 (DDEF2), mRNA. |
| *C3orf59* | 227599_at | 0.0082529 | 0.357325 | -1.25 | Hs.151443 | 3q29 | chromosome 3 open reading frame 59 (C3orf59), mRNA. |
| *ZNF275* | 225383_at | 0.0083906 | 0.357325 | -1.25 | Hs.348963 | Xq28 | zinc finger protein 275 (ZNF275), mRNA. |
| *FNBP1* | 212288_at | 0.0084116 | 0.357325 | -1.25 | Hs.189409 | 9q34 | formin binding protein 1 (FNBP1), mRNA. |
| *SIPA1L1* | 237032_x_at | 0.008449 | 0.357325 | -1.25 | Hs.654657 | 14q24.2 | Signal-induced proliferation-associated 1 like 1 |
| *CHD1* | 235791_x_at | 0.0091495 | 0.3669665 | -1.25 | Hs.643465 | 5q15-q21 | chromodomain helicase DNA binding protein 1 (CHD1), mRNA. |
| *BCHE* | 205433_at | 0.0091555 | 0.3669665 | -1.25 | Hs.420483 | 3q26.1-q26.2 | butyrylcholinesterase (BCHE), mRNA. |
| *PGM2* | 223738_s_at | 0.0096361 | 0.3735558 | -1.25 | Hs.23363 | 4p14 | phosphoglucomutase 2 (PGM2), mRNA. |
| *RANBP5* | 211955_at | 0.0099256 | 0.3735558 | -1.25 | Hs.643743 | 13q32.2 | RAN binding protein 5 (RANBP5), mRNA. |
| *TPP1* | 200742_s_at | 0.0000213 | 0.1504141 | -1.4285714 | Hs.523454 | 11p15 | tripeptidyl peptidase I (TPP1), mRNA. |
| *RBAK* | 228571_at | 0.0000817 | 0.2220164 | -1.4285714 | Hs.592827 | 7p22.1 | RB-associated KRAB zinc finger (RBAK), mRNA. |
|  | 213743_at | 0.0001475 | 0.233361 | -1.4285714 | Hs.666595 | 2 | Transcribed locus |
| *STK3* | 204068_at | 0.0002071 | 0.233361 | -1.4285714 | Hs.492333 | 8q22.2 | serine/threonine kinase 3 (STE20 homolog, yeast) (STK3), mRNA. |
| *LOC26010* | 222154_s_at | 0.0003038 | 0.2454125 | -1.4285714 | Hs.120323 | 2q33.1 | viral DNA polymerase-transactivated protein 6 (LOC26010), mRNA. |
| *EFEMP1* | 201843_s_at | 0.0004109 | 0.2680312 | -1.4285714 | Hs.76224 | 2p16 | EGF-containing fibulin-like extracellular matrix protein 1 (EFEMP1), transcript variant 3, mRNA. |
| *HSPA14* | 227650_at | 0.0004314 | 0.2680312 | -1.4285714 | Hs.534169 | 10p13 | Heat shock 70kDa protein 14 |
| *PRDM2* | 203057_s_at | 0.0007026 | 0.305613 | -1.4285714 | Hs.371823 | 1p36.21 | PR domain containing 2, with ZNF domain (PRDM2), transcript variant 3, mRNA. |
| *DCLK1* | 205399_at | 0.0007223 | 0.3063274 | -1.4285714 | Hs.507755 | 13q13 | doublecortin-like kinase 1 (DCLK1), mRNA. |
| *ZNF655* | 225945_at | 0.0008195 | 0.3119901 | -1.4285714 | Hs.521064 | 7q22.1 | zinc finger protein 655 (ZNF655), transcript variant 3, mRNA. |
|  | 229114_at | 0.0011877 | 0.357325 | -1.4285714 | Hs.632864 | 4 | CDNA clone IMAGE:4801326 |
| *TBC1D23* | 225121_at | 0.0012386 | 0.357325 | -1.4285714 | Hs.477003 | 3q12.1-q12.2 | TBC1 domain family, member 23 |
| *STX12* | 212112_s_at | 0.0012815 | 0.357325 | -1.4285714 | Hs.523855 | 1p35-p34.1 | syntaxin 12 (STX12), mRNA. |
| *XRRA1* | 226739_at | 0.0014318 | 0.357325 | -1.4285714 | Hs.370145 | 11 | PREDICTED: X-ray radiation resistance associated 1, transcript variant 1 (XRRA1), mRNA. |
| *SPAG9* | 225339_at | 0.0014786 | 0.357325 | -1.4285714 | Hs.593620 | 17q21.33 | Sperm associated antigen 9 |
| *OSBPL3* | 209626_s_at | 0.0015281 | 0.357325 | -1.4285714 | Hs.520259 | 7p15 | oxysterol binding protein-like 3 (OSBPL3), transcript variant 4, mRNA. |
| *PLAGL2* | 202925_s_at | 0.0017175 | 0.357325 | -1.4285714 | Hs.154104 | 20q11.21 | pleiomorphic adenoma gene-like 2 (PLAGL2), mRNA. |
| *MYH10* | 212372_at | 0.0017723 | 0.357325 | -1.4285714 | Hs.16355 | 17p13 | myosin, heavy chain 10, non-muscle (MYH10), mRNA. |
| *SPAG9* | 212470_at | 0.0018761 | 0.357325 | -1.4285714 | Hs.463439 | 17q21.33 | sperm associated antigen 9 (SPAG9), mRNA. |
| *NFAT5* | 224984_at | 0.0019327 | 0.357325 | -1.4285714 | Hs.371987 | 16q22.1 | nuclear factor of activated T-cells 5, tonicity-responsive (NFAT5), transcript variant 3, mRNA. |
| *CXorf15* | 227520_at | 0.0023998 | 0.357325 | -1.4285714 | Hs.555961 | Xp22.2 | Chromosome X open reading frame 15 |
| *ZNF764* | 57516_at | 0.002418 | 0.357325 | -1.4285714 | Hs.132227 | 16p11.2 | Zinc finger protein 764 |
| *SEC31A* | 215009_s_at | 0.0024688 | 0.357325 | -1.4285714 | Hs.370024 | 4q21.22 | SEC31 homolog A (S. cerevisiae) |
| *RCOR1* | 212612_at | 0.0024764 | 0.357325 | -1.4285714 | Hs.510521 | 14q32.31-q32.32 | REST corepressor 1 (RCOR1), mRNA. |
| *LOC401504* | 226635_at | 0.0025027 | 0.357325 | -1.4285714 | Hs.446271 | 9p13.2 | Hypothetical gene supported by AK091718 |
| *DSE* | 218854_at | 0.0031671 | 0.357325 | -1.4285714 | Hs.486292 | 6q22 | dermatan sulfate epimerase (DSE), transcript variant 2, mRNA. |
|  | 235733_at | 0.0034004 | 0.357325 | -1.4285714 | Hs.659021 | 3 | Transcribed locus |
| *LOC727858* | 223327_x_at | 0.0038864 | 0.357325 | -1.4285714 |  | 15 | PREDICTED: similar to Dynamin-1 (D100) (Dynamin, brain) (B-dynamin) (LOC727858), mRNA. |
| *ZNF30* | 232014_at | 0.0039952 | 0.357325 | -1.4285714 | Hs.657402 | 19q13.11 | zinc finger protein 30 (ZNF30), mRNA. |
|  | 227458_at | 0.0042444 | 0.357325 | -1.4285714 | Hs.655937 | 9 | Transcribed locus |
| *ARL13B* | 228201_at | 0.0044143 | 0.357325 | -1.4285714 | Hs.533086 | 3q11.2 | ADP-ribosylation factor-like 13B (ARL13B), transcript variant 2, mRNA. |
| *NDUFA5* | 201304_at | 0.0045484 | 0.357325 | -1.4285714 | Hs.651219 | 7q32 | NADH dehydrogenase (ubiquinone) 1 alpha subcomplex, 5, 13kDa (NDUFA5), nuclear gene encoding mitochondrial protein, mRNA. |
| *RP5-1022P6.2* | 224835_at | 0.004627 | 0.357325 | -1.4285714 | Hs.636359 | 20p12.3 | Hypothetical protein KIAA1434 |
| *GKAP1* | 234192_s_at | 0.0046463 | 0.357325 | -1.4285714 | Hs.522255 | 9q21.32 | G kinase anchoring protein 1 (GKAP1), mRNA. |
|  | 227554_at | 0.0047666 | 0.357325 | -1.4285714 | Hs.31474 | 7 | MRNA; cDNA DKFZp686I18116 (from clone DKFZp686I18116) |
| *ELF4* | 31845_at | 0.004809 | 0.357325 | -1.4285714 | Hs.271940 | Xq26 | E74-like factor 4 (ets domain transcription factor) |
|  | 227755_at | 0.0049335 | 0.357325 | -1.4285714 | Hs.356481 | 17 | CDNA clone IMAGE:4077090 |
| *PNRC2* | 217779_s_at | 0.0049489 | 0.357325 | -1.4285714 | Hs.512636 | 1p36.11 | proline-rich nuclear receptor coactivator 2 (PNRC2), mRNA. |
| *AKAP13* | 221718_s_at | 0.0049564 | 0.357325 | -1.4285714 | Hs.459211 | 15q24-q25 | A kinase (PRKA) anchor protein 13 (AKAP13), transcript variant 2, mRNA. |
| *TNPO1* | 209226_s_at | 0.0053152 | 0.357325 | -1.4285714 | Hs.482497 | 5q13.2 | transportin 1 (TNPO1), transcript variant 2, mRNA. |
| *YOD1* | 227309_at | 0.0053875 | 0.357325 | -1.4285714 | Hs.567533 | 1q32.1 | YOD1 OTU deubiquinating enzyme 1 homolog (S. cerevisiae) (YOD1), mRNA. |
| *SLFN11* | 226743_at | 0.0054994 | 0.357325 | -1.4285714 | Hs.462829 | 17q12 | schlafen family member 11 (SLFN11), mRNA. |
| *RAPH1* | 225186_at | 0.0055996 | 0.357325 | -1.4285714 | Hs.471162 | 2q33 | Ras association (RalGDS/AF-6) and pleckstrin homology domains 1 (RAPH1), transcript variant 1, mRNA. |
| *ANKRD12* | 212286_at | 0.0056841 | 0.357325 | -1.4285714 | Hs.464585 | 18p11.22 | ankyrin repeat domain 12 (ANKRD12), transcript variant 2, mRNA. |
| *POGK* | 218229_s_at | 0.0057144 | 0.357325 | -1.4285714 | Hs.432752 | 1q24.1 | pogo transposable element with KRAB domain (POGK), mRNA. |
| *BNC1* | 1552487_a_at | 0.0058374 | 0.357325 | -1.4285714 | Hs.459153 | 15q25.2 | basonuclin 1 (BNC1), mRNA. |
| *LHFP* | 218656_s_at | 0.005894 | 0.357325 | -1.4285714 | Hs.507798 | 13q12 | lipoma HMGIC fusion partner (LHFP), mRNA. |
| *ARL1* | 201657_at | 0.0059158 | 0.357325 | -1.4285714 | Hs.372616 | 12q23.2 | ADP-ribosylation factor-like 1 (ARL1), mRNA. |
| *LOC387647* | 228786_at | 0.0061258 | 0.357325 | -1.4285714 |  | 10 | PREDICTED: hypothetical gene supported by BC014163 (LOC387647), mRNA. |
| *ZFP28* | 231943_at | 0.0061478 | 0.357325 | -1.4285714 | Hs.14794 | 19q13.43 | zinc finger protein 28 homolog (mouse) (ZFP28), mRNA. |
| *TLE4* | 204872_at | 0.0061715 | 0.357325 | -1.4285714 | Hs.444213 | 9q21.31 | Transducin-like enhancer of split 4 (E(sp1) homolog, Drosophila) |
|  | 228315_at | 0.006187 | 0.357325 | -1.4285714 | Hs.371609 | 3 | CDNA clone IMAGE:5261213 |
| *SLC25A33* | 223296_at | 0.0063254 | 0.357325 | -1.4285714 | Hs.568613 | 1p36.22 | solute carrier family 25, member 33 (SLC25A33), mRNA. |
| *ASXL2* | 226251_at | 0.006329 | 0.357325 | -1.4285714 | Hs.655514 | 2p24.1 | Additional sex combs like 2 (Drosophila) |
| *EPAS1* | 200878_at | 0.0064805 | 0.357325 | -1.4285714 | Hs.468410 | 2p21-p16 | endothelial PAS domain protein 1 (EPAS1), mRNA. |
| *TOR1AIP1* | 212408_at | 0.006482 | 0.357325 | -1.4285714 | Hs.496459 | 1q24.2 | torsin A interacting protein 1 (TOR1AIP1), mRNA. |
| *C14orf118* | 229520_s_at | 0.0065931 | 0.357325 | -1.4285714 | Hs.410231 | 14q22.1-q24.3 | Chromosome 14 open reading frame 118 |
| *TLE1* | 228284_at | 0.0066652 | 0.357325 | -1.4285714 | Hs.197320 | 9q21.32 | Transducin-like enhancer of split 1 (E(sp1) homolog, Drosophila) |
| *RUNX2* | 232231_at | 0.0067188 | 0.357325 | -1.4285714 | Hs.535845 | 6p21 | runt-related transcription factor 2 (RUNX2), transcript variant 2, mRNA. |
| *C9orf72* | 1553133_at | 0.0067585 | 0.357325 | -1.4285714 | Hs.493639 | 9p21.2 | chromosome 9 open reading frame 72 (C9orf72), transcript variant 2, mRNA. |
| *SLC2A3* | 222088_s_at | 0.0068 | 0.357325 | -1.4285714 | Hs.419240 | 12p13.3 | solute carrier family 2 (facilitated glucose transporter), member 3 (SLC2A3), mRNA. |
| *HNRPDL* | 201993_x_at | 0.0070616 | 0.357325 | -1.4285714 |  | 4q13-q21 | heterogeneous nuclear ribonucleoprotein D-like (HNRPDL), transcript variant 3, transcribed RNA. |
| *RYBP* | 201845_s_at | 0.0071578 | 0.357325 | -1.4285714 | Hs.7910 | 3p13 | RING1 and YY1 binding protein (RYBP), mRNA. |
| *TPCN1* | 217914_at | 0.00722 | 0.357325 | -1.4285714 | Hs.524763 | 12q24.13 | Two pore segment channel 1 |
| *RAPGEF1* | 225738_at | 0.0072831 | 0.357325 | -1.4285714 | Hs.127897 | 9q34.3 | Rap guanine nucleotide exchange factor (GEF) 1 (RAPGEF1), transcript variant 1, mRNA. |
| *PACSIN2* | 201651_s_at | 0.0075812 | 0.357325 | -1.4285714 | Hs.162877 | 22q13.2-q13.33 | protein kinase C and casein kinase substrate in neurons 2 (PACSIN2), mRNA. |
| *ZNF627* | 224492_s_at | 0.0077055 | 0.357325 | -1.4285714 | Hs.526665 | 19p13.2 | zinc finger protein 627 (ZNF627), mRNA. |
| *ACVR2A* | 205327_s_at | 0.0078633 | 0.357325 | -1.4285714 | Hs.470174 | 2q22.3 | activin A receptor, type IIA (ACVR2A), mRNA. |
| *NFE2L2* | 201146_at | 0.0079863 | 0.357325 | -1.4285714 | Hs.155396 | 2q31 | nuclear factor (erythroid-derived 2)-like 2 (NFE2L2), mRNA. |
|  | 227772_at | 0.0080098 | 0.357325 | -1.4285714 | Hs.633030 | 6 | Transcribed locus |
| *KIAA0888* | 235048_at | 0.008086 | 0.357325 | -1.4285714 | Hs.91662 | 5q13.3 | KIAA0888 protein |
| *CXorf15* | 1557954_at | 0.0083411 | 0.357325 | -1.4285714 | Hs.555961 | Xp22.2 | Chromosome X open reading frame 15 |
| *NRG1* | 206343_s_at | 0.0085683 | 0.357325 | -1.4285714 | Hs.453951 | 8p21-p12 | neuregulin 1 (NRG1), transcript variant HRG-beta3, mRNA. |
| *PTTG3* | 208511_at | 0.008594 | 0.357325 | -1.4285714 |  | 8q13.1 | pituitary tumor-transforming 3 (PTTG3) on chromosome 8. |
| *CTNNAL1* | 202468_s_at | 0.0089972 | 0.3660156 | -1.4285714 | Hs.58488 | 9q31.2 | catenin (cadherin-associated protein), alpha-like 1 (CTNNAL1), mRNA. |
| *RPL37* | 224767_at | 0.0090721 | 0.3669665 | -1.4285714 | Hs.80545 | 5p13 | Ribosomal protein L37 |
| *PIK3CD* | 203879_at | 0.0092088 | 0.367572 | -1.4285714 | Hs.518451 | 1p36.2 | phosphoinositide-3-kinase, catalytic, delta polypeptide (PIK3CD), mRNA. |
| *FLJ36874* | 225468_at | 0.0094862 | 0.3730549 | -1.4285714 | Hs.591960 | 11q12.1 | FLJ36874 protein (FLJ36874), mRNA. |
| *C16orf72* | 225183_at | 0.0094954 | 0.3730549 | -1.4285714 | Hs.221497 | 16p13.2 | chromosome 16 open reading frame 72 (C16orf72), mRNA. |
| *C1orf63* | 209007_s_at | 0.0095322 | 0.373122 | -1.4285714 | Hs.259412 | 1p36.13-p35.1 | chromosome 1 open reading frame 63 (C1orf63), mRNA. |
| *ISOC1* | 218170_at | 0.0097645 | 0.3735558 | -1.4285714 | Hs.483296 | 5q22.1-q33.3 | isochorismatase domain containing 1 (ISOC1), mRNA. |
| *FAM83D* | 225687_at | 0.0099338 | 0.3735558 | -1.4285714 | Hs.472716 | 20q11.22-q12 | family with sequence similarity 83, member D (FAM83D), mRNA. |
| *C14orf147* | 212460_at | 0.0099403 | 0.3735558 | -1.4285714 | Hs.269909 | 14q13.1 | chromosome 14 open reading frame 147 (C14orf147), mRNA. |
| *USP54* | 227334_at | 0.0002201 | 0.233361 | -1.6666667 | Hs.657355 | 10q22.2 | ubiquitin specific peptidase 54 (USP54), mRNA. |
| *USP38* | 223289_s_at | 0.000235 | 0.2345024 | -1.6666667 | Hs.480848 | 4q31.1 | ubiquitin specific peptidase 38 (USP38), mRNA. |
| *KBTBD9* | 229310_at | 0.0002604 | 0.2454125 | -1.6666667 | Hs.130593 | 2 | PREDICTED: kelch repeat and BTB (POZ) domain containing 9 (KBTBD9), mRNA. |
| *SLC39A14* | 212110_at | 0.0003398 | 0.2620167 | -1.6666667 | Hs.491232 | 8p21.3 | solute carrier family 39 (zinc transporter), member 14 (SLC39A14), mRNA. |
| *NUFIP2* | 224958_at | 0.0003736 | 0.2640729 | -1.6666667 | Hs.462598 | 17q11.2 | nuclear fragile X mental retardation protein interacting protein 2 (NUFIP2), mRNA. |
| *FAM53A* | 1569139_s_at | 0.0005803 | 0.2983094 | -1.6666667 | Hs.143314 | 4p16.3 | family with sequence similarity 53, member A (FAM53A), mRNA. |
| *MAN2A1* | 235103_at | 0.0006133 | 0.305613 | -1.6666667 | Hs.432822 | 5q21-q22 | Mannosidase, alpha, class 2A, member 1 |
| *EGFR* | 201984_s_at | 0.0006421 | 0.305613 | -1.6666667 | Hs.488293 | 7p12 | epidermal growth factor receptor (erythroblastic leukemia viral (v-erb-b) oncogene homolog, avian) (EGFR), transcript variant 1, mRNA. |
| *SCARA3* | 219416_at | 0.000846 | 0.3119901 | -1.6666667 | Hs.128856 | 8p21 | scavenger receptor class A, member 3 (SCARA3), transcript variant 1, mRNA. |
| *BGLAP* | 206956_at | 0.0013331 | 0.357325 | -1.6666667 | Hs.654541 | 1q25-q31 | bone gamma-carboxyglutamate (gla) protein (osteocalcin) (BGLAP), mRNA. |
| *RBM24* | 235004_at | 0.0016148 | 0.357325 | -1.6666667 | Hs.519904 | 6p22.3 | RNA binding motif protein 24 (RBM24), mRNA. |
| *LOC153222* | 225957_at | 0.0016418 | 0.357325 | -1.6666667 | Hs.484195 | 5q35.2 | adult retina protein (LOC153222), mRNA. |
| *EPSTI1* | 227609_at | 0.001689 | 0.357325 | -1.6666667 | Hs.546467 | 13q13.3 | epithelial stromal interaction 1 (breast) (EPSTI1), transcript variant 2, mRNA. |
|  | 231907_at | 0.0019315 | 0.357325 | -1.6666667 | Hs.159472 | 1 | CDNA FLJ31718 fis, clone NT2RI2006647 |
| *LATS2* | 227013_at | 0.001934 | 0.357325 | -1.6666667 | Hs.78960 | 13q11-q12 | LATS, large tumor suppressor, homolog 2 (Drosophila) (LATS2), mRNA. |
| *C1orf25* | 233750_s_at | 0.0022732 | 0.357325 | -1.6666667 | Hs.591488 | 1q25.2 | chromosome 1 open reading frame 25 (C1orf25), mRNA. |
| *KIAA0286* | 212621_at | 0.0023078 | 0.357325 | -1.6666667 | Hs.591040 | 12q13.3 | KIAA0286 protein (KIAA0286), mRNA. |
| *COBLL1* | 203642_s_at | 0.002391 | 0.357325 | -1.6666667 | Hs.470457 | 2q24.3 | COBL-like 1 (COBLL1), mRNA. |
| *SLC2A3* | 202499_s_at | 0.002459 | 0.357325 | -1.6666667 | Hs.419240 | 12p13.3 | solute carrier family 2 (facilitated glucose transporter), member 3 (SLC2A3), mRNA. |
| *THRAP4* | 213043_s_at | 0.0026853 | 0.357325 | -1.6666667 | Hs.462983 | 17q21.1 | thyroid hormone receptor associated protein 4 (THRAP4), transcript variant 2, mRNA. |
| *UBTD2* | 224827_at | 0.0028662 | 0.357325 | -1.6666667 | Hs.179852 | 5q35.1 | ubiquitin domain containing 2 (UBTD2), mRNA. |
| *GFPT2* | 205100_at | 0.0030804 | 0.357325 | -1.6666667 | Hs.30332 | 5q34-q35 | glutamine-fructose-6-phosphate transaminase 2 (GFPT2), mRNA. |
| *EGFR* | 201983_s_at | 0.0031384 | 0.357325 | -1.6666667 | Hs.488293 | 7p12 | epidermal growth factor receptor (erythroblastic leukemia viral (v-erb-b) oncogene homolog, avian) (EGFR), transcript variant 1, mRNA. |
| *GALNT12* | 218885_s_at | 0.0032672 | 0.357325 | -1.6666667 | Hs.47099 | 9q22.33 | UDP-N-acetyl-alpha-D-galactosamine:polypeptide N-acetylgalactosaminyltransferase 12 (GalNAc-T12) (GALNT12), mRNA. |
| *RUNX1* | 210365_at | 0.0033092 | 0.357325 | -1.6666667 | Hs.149261 | 21q22.3 | Runt-related transcription factor 1 (acute myeloid leukemia 1; aml1 oncogene) |
| *WSB1* | 210561_s_at | 0.0036717 | 0.357325 | -1.6666667 | Hs.446017 | 17q11.1 | WD repeat and SOCS box-containing 1 (WSB1), transcript variant 2, mRNA. |
| *SVEP1* | 213247_at | 0.0038739 | 0.357325 | -1.6666667 | Hs.522334 | 9q32 | sushi, von Willebrand factor type A, EGF and pentraxin domain containing 1 (SVEP1), mRNA. |
| *KLHL15* | 226370_at | 0.0041619 | 0.357325 | -1.6666667 | Hs.495854 | Xp22.1-p21 | Kelch-like 15 (Drosophila) |
| *OGFR* | 210443_x_at | 0.0042069 | 0.357325 | -1.6666667 | Hs.67896 | 20q13.3 | opioid growth factor receptor (OGFR), mRNA. |
| *IDI1* | 204615_x_at | 0.0042217 | 0.357325 | -1.6666667 | Hs.283652 | 10p15.3 | isopentenyl-diphosphate delta isomerase 1 (IDI1), mRNA. |
| *PDLIM5* | 216804_s_at | 0.0042392 | 0.357325 | -1.6666667 | Hs.480311 | 4q22 | PDZ and LIM domain 5 (PDLIM5), transcript variant 2, mRNA. |
| *FZD8* | 224325_at | 0.0043368 | 0.357325 | -1.6666667 | Hs.302634 | 10p11.21 | frizzled homolog 8 (Drosophila) (FZD8), mRNA. |
| *EEA1* | 204841_s_at | 0.0044583 | 0.357325 | -1.6666667 | Hs.567367 | 12q22 | early endosome antigen 1 (EEA1), mRNA. |
| *KLHL21* | 203068_at | 0.0046312 | 0.357325 | -1.6666667 | Hs.7764 | 1p36.31 | kelch-like 21 (Drosophila) (KLHL21), mRNA. |
| *CA12* | 215867_x_at | 0.0046877 | 0.357325 | -1.6666667 | Hs.210995 | 15q22 | carbonic anhydrase XII (CA12), transcript variant 2, mRNA. |
| *IDI1* | 208881_x_at | 0.0047392 | 0.357325 | -1.6666667 | Hs.283652 | 10p15.3 | isopentenyl-diphosphate delta isomerase 1 (IDI1), mRNA. |
| *ZNF295* | 233952_s_at | 0.0050226 | 0.357325 | -1.6666667 | Hs.434947 | 21q22.3 | zinc finger protein 295 (ZNF295), mRNA. |
| *FZD8* | 227405_s_at | 0.0051177 | 0.357325 | -1.6666667 | Hs.302634 | 10p11.21 | frizzled homolog 8 (Drosophila) (FZD8), mRNA. |
| *PPM1D* | 204566_at | 0.0052724 | 0.357325 | -1.6666667 | Hs.591184 | 17q23.2 | protein phosphatase 1D magnesium-dependent, delta isoform (PPM1D), mRNA. |
| *ZBTB5* | 203026_at | 0.0052954 | 0.357325 | -1.6666667 | Hs.161276 | 9p13.2 | zinc finger and BTB domain containing 5 (ZBTB5), mRNA. |
| *WDR20* | 1554549_a_at | 0.0056228 | 0.357325 | -1.6666667 | Hs.36859 | 14q32.31 | WD repeat domain 20 |
| *ARHGAP27* | 225618_at | 0.0057126 | 0.357325 | -1.6666667 | Hs.569809 | 17q21.31 | Rho GTPase activating protein 27 (ARHGAP27), mRNA. |
|  | 227200_at | 0.0058902 | 0.357325 | -1.6666667 | Hs.652466 | 1 | Transcribed locus |
| *ARHGAP23* | 226638_at | 0.0060873 | 0.357325 | -1.6666667 | Hs.374446 | 17q12 | Rho GTPase activating protein 23 |
| *MEIS1* | 204069_at | 0.0061009 | 0.357325 | -1.6666667 | Hs.526754 | 2p14-p13 | Meis homeobox 1 (MEIS1), mRNA. |
| *KLF9* | 203542_s_at | 0.0061468 | 0.357325 | -1.6666667 | Hs.150557 | 9q13 | Kruppel-like factor 9 (KLF9), mRNA. |
| *CCNYL1* | 228810_at | 0.0062321 | 0.357325 | -1.6666667 | Hs.471234 | 2q33.3 | cyclin Y-like 1 (CCNYL1), mRNA. |
|  | 225133_at | 0.0062724 | 0.357325 | -1.6666667 | Hs.598860 | 4 | Transcript ch138 [human, RF1,RF48 stomach cancer cell lines, mRNA, 235 nt] |
| *LDLR* | 202068_s_at | 0.00629 | 0.357325 | -1.6666667 | Hs.213289 | 19p13.3 | low density lipoprotein receptor (familial hypercholesterolemia) (LDLR), mRNA. |
| *TIPARP* | 212665_at | 0.0063532 | 0.357325 | -1.6666667 | Hs.12813 | 3q25.31 | TCDD-inducible poly(ADP-ribose) polymerase (TIPARP), mRNA. |
| *EDG3* | 228176_at | 0.0064837 | 0.357325 | -1.6666667 | Hs.585118 | 9q22.1-q22.2 | endothelial differentiation, sphingolipid G-protein-coupled receptor, 3 (EDG3), mRNA. |
| *C1QTNF5* | 223499_at | 0.0064909 | 0.357325 | -1.6666667 | Hs.632102 | 11q23.3 | C1q and tumor necrosis factor related protein 5 (C1QTNF5), mRNA. |
| *IL6* | 205207_at | 0.0066491 | 0.357325 | -1.6666667 | Hs.654458 | 7p21 | interleukin 6 (interferon, beta 2) (IL6), mRNA. |
| *HSPB8* | 221667_s_at | 0.0066717 | 0.357325 | -1.6666667 | Hs.400095 | 12q24.23 | heat shock 22kDa protein 8 (HSPB8), mRNA. |
| *SIRT1* | 218878_s_at | 0.0067017 | 0.357325 | -1.6666667 | Hs.369779 | 10q21.3 | sirtuin (silent mating type information regulation 2 homolog) 1 (S. cerevisiae) (SIRT1), mRNA. |
| *FAM110B* | 228790_at | 0.0068175 | 0.357325 | -1.6666667 | Hs.154652 | 8q12.1 | family with sequence similarity 110, member B (FAM110B), mRNA. |
| *ARHGAP17* | 218076_s_at | 0.0068858 | 0.357325 | -1.6666667 | Hs.373793 | 16p12.1 | Rho GTPase activating protein 17 (ARHGAP17), transcript variant 2, mRNA. |
| *FEM1C* | 213341_at | 0.0069145 | 0.357325 | -1.6666667 | Hs.47367 | 5q22 | fem-1 homolog c (C. elegans) (FEM1C), mRNA. |
| *LRRC49* | 219338_s_at | 0.0070687 | 0.357325 | -1.6666667 | Hs.12692 | 15q23 | leucine rich repeat containing 49 (LRRC49), mRNA. |
| *FNIP1* | 228250_at | 0.0071294 | 0.357325 | -1.6666667 | Hs.591273 | 5q23.3 | folliculin interacting protein 1 (FNIP1), transcript variant 2, mRNA. |
| *AXUD1* | 225557_at | 0.007165 | 0.357325 | -1.6666667 | Hs.370950 | 3p22 | AXIN1 up-regulated 1 (AXUD1), mRNA. |
| *ZNF20* | 213916_at | 0.0073065 | 0.357325 | -1.6666667 | Hs.512823 | 19p13.3-p13.2 | zinc finger protein 20 (ZNF20), mRNA. |
| *DAB2* | 210757_x_at | 0.007367 | 0.357325 | -1.6666667 | Hs.481980 | 5p13 | disabled homolog 2, mitogen-responsive phosphoprotein (Drosophila) (DAB2), mRNA. |
| *TNIK* | 213109_at | 0.0074564 | 0.357325 | -1.6666667 | Hs.34024 | 3q26.2-q26.31 | TRAF2 and NCK interacting kinase |
| *SPNS1* | 223173_at | 0.0075754 | 0.357325 | -1.6666667 | Hs.632181 | 16p11.2 | spinster homolog 1 (Drosophila) (SPNS1), mRNA. |
| *CLDN1* | 218182_s_at | 0.0076521 | 0.357325 | -1.6666667 | Hs.439060 | 3q28-q29 | claudin 1 (CLDN1), mRNA. |
| *ARID5B* | 241969_at | 0.0076786 | 0.357325 | -1.6666667 | Hs.535297 | 10q21.2 | AT rich interactive domain 5B (MRF1-like) |
| *VGLL3* | 227399_at | 0.0077634 | 0.357325 | -1.6666667 | Hs.435013 | 3p12.1 | vestigial like 3 (Drosophila) (VGLL3), mRNA. |
| *ZNF92* | 235170_at | 0.0079039 | 0.357325 | -1.6666667 | Hs.9521 | 7q11.21 | zinc finger protein 92 (ZNF92), transcript variant 1, mRNA. |
| *DCP2* | 235258_at | 0.0080856 | 0.357325 | -1.6666667 | Hs.443875 | 5q22.2 | DCP2 decapping enzyme homolog (S. cerevisiae) (DCP2), mRNA. |
| *OBFC2A* | 219334_s_at | 0.008118 | 0.357325 | -1.6666667 | Hs.591610 | 2q32.3 | Oligonucleotide/oligosaccharide-binding fold containing 2A |
| *CDC42EP3* | 209286_at | 0.0082239 | 0.357325 | -1.6666667 | Hs.369574 | 2p21 | CDC42 effector protein (Rho GTPase binding) 3 (CDC42EP3), mRNA. |
| *FZD7* | 203705_s_at | 0.0082574 | 0.357325 | -1.6666667 | Hs.173859 | 2q33 | frizzled homolog 7 (Drosophila) (FZD7), mRNA. |
| *NRP2* | 1555468_at | 0.0083033 | 0.357325 | -1.6666667 | Hs.471200 | 2q33.3 | Neuropilin 2 |
| *DNAJB9* | 202843_at | 0.0083656 | 0.357325 | -1.6666667 | Hs.6790 | 7q31 | DnaJ (Hsp40) homolog, subfamily B, member 9 (DNAJB9), mRNA. |
|  | 217523_at | 0.0085104 | 0.357325 | -1.6666667 | Hs.668083 | 11 | Transcribed locus |
| *MCAM* | 209087_x_at | 0.0085139 | 0.357325 | -1.6666667 | Hs.599039 | 11q23.3 | melanoma cell adhesion molecule (MCAM), mRNA. |
| *CIDEC* | 1554839_at | 0.008588 | 0.357325 | -1.6666667 | Hs.635072 | 3p25.3 | Cell death-inducing DFFA-like effector c |
| *BCL6* | 203140_at | 0.0087072 | 0.3587496 | -1.6666667 | Hs.478588 | 3q27 | B-cell CLL/lymphoma 6 (zinc finger protein 51) (BCL6), transcript variant 1, mRNA. |
| *KCTD12* | 212192_at | 0.0091647 | 0.3669665 | -1.6666667 |  | 13q22.3 | potassium channel tetramerisation domain containing 12 (KCTD12), mRNA. |
| *KIAA0247* | 202181_at | 0.009172 | 0.3669665 | -1.6666667 | Hs.440025 | 14q24.1 | KIAA0247 (KIAA0247), mRNA. |
| *IRS2* | 209184_s_at | 0.0092847 | 0.3688657 | -1.6666667 | Hs.442344 | 13q34 | insulin receptor substrate 2 (IRS2), mRNA. |
| *CYLD* | 221903_s_at | 0.0095001 | 0.3730549 | -1.6666667 | Hs.578973 | 16q12.1 | cylindromatosis (turban tumor syndrome) (CYLD), transcript variant 2, mRNA. |
|  | 236402_at | 0.0000652 | 0.2212106 | -2 | Hs.600998 | 7 | CDNA FLJ42263 fis, clone TKIDN2014570 |
| *LIF* | 205266_at | 0.0001832 | 0.233361 | -2 | Hs.2250 | 22q12.2 | leukemia inhibitory factor (cholinergic differentiation factor) (LIF), mRNA. |
|  | 244177_at | 0.0002751 | 0.2454125 | -2 | Hs.508729 | 13 | Transcribed locus |
| *HRH1* | 205579_at | 0.0003577 | 0.2638271 | -2 | Hs.1570 | 3p25 | histamine receptor H1 (HRH1), mRNA. |
| *BACH1* | 204194_at | 0.0003937 | 0.2671491 | -2 | Hs.154276 | 21q22.11 | BTB and CNC homology 1, basic leucine zipper transcription factor 1 (BACH1), transcript variant 2, mRNA. |
| *CBX4* | 227558_at | 0.000473 | 0.2766887 | -2 | Hs.405046 | 17q25.3 | chromobox homolog 4 (Pc class homolog, Drosophila) (CBX4), mRNA. |
| *PRRX1* | 226695_at | 0.0006828 | 0.305613 | -2 | Hs.283416 | 1q24 | paired related homeobox 1 (PRRX1), transcript variant pmx-1b, mRNA. |
| *ZC3H12C* | 231899_at | 0.0007007 | 0.305613 | -2 | Hs.376289 | 11q22.3 | Zinc finger CCCH-type containing 12C |
| *KSR1* | 235252_at | 0.0007762 | 0.3119901 | -2 | Hs.133534 | 17q11.1 | Kinase suppressor of ras 1 |
| *ID2* | 201566_x_at | 0.0008276 | 0.3119901 | -2 | Hs.180919 | 2p25 | Inhibitor of DNA binding 2, dominant negative helix-loop-helix protein |
| *KIAA1217* | 231807_at | 0.0008282 | 0.3119901 | -2 | Hs.445885 | 10p12.1 | KIAA1217 |
| *SGMS2* | 242963_at | 0.0014254 | 0.357325 | -2 | Hs.595423 | 4q25 | sphingomyelin synthase 2 (SGMS2), mRNA. |
| *MCL1* | 200798_x_at | 0.0016531 | 0.357325 | -2 | Hs.632486 | 1q21 | myeloid cell leukemia sequence 1 (BCL2-related) (MCL1), transcript variant 2, mRNA. |
| *HIVEP2* | 212641_at | 0.001681 | 0.357325 | -2 | Hs.510172 | 6q23-q24 | human immunodeficiency virus type I enhancer binding protein 2 (HIVEP2), mRNA. |
| *FAS* | 204781_s_at | 0.0019131 | 0.357325 | -2 | Hs.244139 | 10q24.1 | Fas (TNF receptor superfamily, member 6) (FAS), transcript variant 6, mRNA. |
| *CAP2* | 212551_at | 0.00201 | 0.357325 | -2 | Hs.132902 | 6p22.3 | CAP, adenylate cyclase-associated protein, 2 (yeast) (CAP2), mRNA. |
| *KITLG* | 226534_at | 0.0020551 | 0.357325 | -2 | Hs.1048 | 12q22 | KIT ligand (KITLG), transcript variant a, mRNA. |
| *ASCC3* | 231269_at | 0.0022457 | 0.357325 | -2 | Hs.486031 | 6q16.1-q16.3 | activating signal cointegrator 1 complex subunit 3 (ASCC3), transcript variant 2, mRNA. |
| *IER5* | 218611_at | 0.0026493 | 0.357325 | -2 | Hs.15725 | 1q25.3 | immediate early response 5 (IER5), mRNA. |
| *HBEGF* | 38037_at | 0.0027689 | 0.357325 | -2 | Hs.799 | 5q23 | heparin-binding EGF-like growth factor (HBEGF), mRNA. |
| *PPP3CC* | 32541_at | 0.003025 | 0.357325 | -2 | Hs.655661 | 8p21.3 | Protein phosphatase 3 (formerly 2B), catalytic subunit, gamma isoform |
| *NUAK2* | 220987_s_at | 0.003186 | 0.357325 | -2 | Hs.497512 | 1q32.1 | NUAK family, SNF1-like kinase, 2 (NUAK2), mRNA. |
| *BAMBI* | 203304_at | 0.003287 | 0.357325 | -2 | Hs.533336 | 10p12.3-p11.2 | BMP and activin membrane-bound inhibitor homolog (Xenopus laevis) (BAMBI), mRNA. |
| *KIAA1344* | 226747_at | 0.0033802 | 0.357325 | -2 | Hs.532609 | 14q22.1 | KIAA1344 (KIAA1344), mRNA. |
| *HIVEP2* | 212642_s_at | 0.0034155 | 0.357325 | -2 | Hs.510172 | 6q23-q24 | human immunodeficiency virus type I enhancer binding protein 2 (HIVEP2), mRNA. |
| *LOC401074* | 1559826_a_at | 0.0034323 | 0.357325 | -2 |  | 3 | PREDICTED: hypothetical LOC401074 (LOC401074), mRNA. |
| *EFNB2* | 202668_at | 0.0035807 | 0.357325 | -2 | Hs.149239 | 13q33 | ephrin-B2 (EFNB2), mRNA. |
| *ZNF79* | 214138_at | 0.0037969 | 0.357325 | -2 | Hs.522399 | 9q34 | zinc finger protein 79 (ZNF79), mRNA. |
| *SPRY2* | 204011_at | 0.0038117 | 0.357325 | -2 | Hs.18676 | 13q31.1 | sprouty homolog 2 (Drosophila) (SPRY2), mRNA. |
| *SCYL1BP1* | 226337_at | 0.0039071 | 0.357325 | -2 | Hs.183702 | 1q24.2 | SCY1-like 1 binding protein 1 (SCYL1BP1), mRNA. |
| *SASH1* | 226022_at | 0.0040022 | 0.357325 | -2 | Hs.193133 | 6q24.3 | SAM and SH3 domain containing 1 (SASH1), mRNA. |
| *ING3* | 205070_at | 0.0040192 | 0.357325 | -2 | Hs.489811 | 7q31 | inhibitor of growth family, member 3 (ING3), transcript variant 1, mRNA. |
| *CRISPLD2* | 221541_at | 0.0040233 | 0.357325 | -2 | Hs.513779 | 16q24.1 | cysteine-rich secretory protein LCCL domain containing 2 (CRISPLD2), mRNA. |
| *MGC5370* | 225160_x_at | 0.0041105 | 0.357325 | -2 | Hs.484551 | 12q14.3 | Hypothetical protein MGC5370 |
| *JUNB* | 201473_at | 0.0042238 | 0.357325 | -2 | Hs.25292 | 19p13.2 | jun B proto-oncogene (JUNB), mRNA. |
| *MAST4* | 225611_at | 0.0044605 | 0.357325 | -2 | Hs.595458 | 5q12.3 | Microtubule associated serine/threonine kinase family member 4 |
| *HDAC9* | 205659_at | 0.0044837 | 0.357325 | -2 | Hs.196054 | 7p21.1 | histone deacetylase 9 (HDAC9), transcript variant 3, mRNA. |
| *KIAA1632* | 227638_at | 0.0045952 | 0.357325 | -2 | Hs.514843 | 18q12.3-q21.1 | KIAA1632 (KIAA1632), mRNA. |
| *ISG20L1* | 219361_s_at | 0.0047801 | 0.357325 | -2 | Hs.436102 | 15q26.1 | interferon stimulated exonuclease gene 20kDa-like 1 (ISG20L1), mRNA. |
| *PLSCR1* | 202430_s_at | 0.0049724 | 0.357325 | -2 | Hs.130759 | 3q23 | phospholipid scramblase 1 (PLSCR1), mRNA. |
| *IL1RL1* | 207526_s_at | 0.0052827 | 0.357325 | -2 | Hs.66 | 2q12 | interleukin 1 receptor-like 1 (IL1RL1), transcript variant 2, mRNA. |
| *GCH1* | 204224_s_at | 0.0054131 | 0.357325 | -2 | Hs.86724 | 14q22.1-q22.2 | GTP cyclohydrolase 1 (dopa-responsive dystonia) (GCH1), transcript variant 4, mRNA. |
| *IL6ST* | 211000_s_at | 0.0054526 | 0.357325 | -2 | Hs.532082 | 5q11 | interleukin 6 signal transducer (gp130, oncostatin M receptor) (IL6ST), transcript variant 2, mRNA. |
| *LMCD1* | 242767_at | 0.0054902 | 0.357325 | -2 | Hs.475353 | 3p26-p24 | LIM and cysteine-rich domains 1 |
| *FOXO3* | 204131_s_at | 0.0055667 | 0.357325 | -2 |  | 6q21 | forkhead box O3 (FOXO3), transcript variant 2, mRNA. |
| *SLC25A27* | 230624_at | 0.0056875 | 0.357325 | -2 | Hs.40510 | 6p11.2-q12 | solute carrier family 25, member 27 (SLC25A27), nuclear gene encoding mitochondrial protein, mRNA. |
|  | 1564378_a_at | 0.0057889 | 0.357325 | -2 | Hs.677321 | 8 | CDNA: FLJ21448 fis, clone COL04473 |
|  | 226893_at | 0.0059413 | 0.357325 | -2 | Hs.159472 | 1 | CDNA FLJ31718 fis, clone NT2RI2006647 |
| *CPEB2* | 226939_at | 0.0060406 | 0.357325 | -2 | Hs.656937 | 4p15.33 | cytoplasmic polyadenylation element binding protein 2 (CPEB2), transcript variant A, mRNA. |
|  | 1558783_at | 0.0061956 | 0.357325 | -2 | Hs.677281 | 6 | CDNA: FLJ21152 fis, clone CAS09594 |
| *TRAF4* | 242473_at | 0.0063644 | 0.357325 | -2 | Hs.8375 | 17q11-q12 | TNF receptor-associated factor 4 (TRAF4), mRNA. |
| *FZD5* | 221245_s_at | 0.0066618 | 0.357325 | -2 | Hs.17631 | 2q33-q34 | frizzled homolog 5 (Drosophila) (FZD5), mRNA. |
| *ZBTB10* | 219312_s_at | 0.0066983 | 0.357325 | -2 | Hs.591868 | 8q13-q21.1 | Zinc finger and BTB domain containing 10 |
| *ASPHD1* | 1553997_a_at | 0.0067191 | 0.357325 | -2 | Hs.655761 | 16p11.2 | aspartate beta-hydroxylase domain containing 1 (ASPHD1), mRNA. |
| *MAST4* | 225613_at | 0.0067387 | 0.357325 | -2 | Hs.595458 | 5q12.3 | Microtubule associated serine/threonine kinase family member 4 |
|  | 228812_at | 0.0071282 | 0.357325 | -2 | Hs.633900 | 2 | Transcribed locus |
| *CSRP2* | 211126_s_at | 0.0074442 | 0.357325 | -2 | Hs.530904 | 12q21.1 | cysteine and glycine-rich protein 2 (CSRP2), mRNA. |
| *SSBP2* | 203787_at | 0.007471 | 0.357325 | -2 | Hs.102735 | 5q14.1 | single-stranded DNA binding protein 2 (SSBP2), mRNA. |
|  | 230083_at | 0.0075163 | 0.357325 | -2 | Hs.600133 | 4 | Transcribed locus |
| *ID4* | 209292_at | 0.0077532 | 0.357325 | -2 | Hs.519601 | 6p22-p21 | Inhibitor of DNA binding 4, dominant negative helix-loop-helix protein |
| *CRISPLD1* | 223475_at | 0.0079743 | 0.357325 | -2 | Hs.436542 | 8q21.11 | cysteine-rich secretory protein LCCL domain containing 1 (CRISPLD1), mRNA. |
| *DRAM* | 218627_at | 0.0080021 | 0.357325 | -2 | Hs.525634 | 12q23.2 | damage-regulated autophagy modulator (DRAM), mRNA. |
| *LOC729436* | 227866_at | 0.0082898 | 0.357325 | -2 | Hs.486886 | 6q25.2 | Hypothetical protein LOC729436 |
|  | 225685_at | 0.0084286 | 0.357325 | -2 | Hs.592787 | 2 | CDNA FLJ31353 fis, clone MESAN2000264 |
| *ZNF670* | 223898_at | 0.0084782 | 0.357325 | -2 | Hs.669893 | 1q44 | zinc finger protein 670 (ZNF670), mRNA. |
| *CDKN2AIP* | 218929_at | 0.008717 | 0.3587496 | -2 | Hs.644077 | 4q35.1 | CDKN2A interacting protein (CDKN2AIP), mRNA. |
| *IL6ST* | 204863_s_at | 0.0089928 | 0.3660156 | -2 | Hs.532082 | 5q11 | interleukin 6 signal transducer (gp130, oncostatin M receptor) (IL6ST), transcript variant 2, mRNA. |
| *NFIL3* | 203574_at | 0.0090657 | 0.3669665 | -2 | Hs.79334 | 9q22 | nuclear factor, interleukin 3 regulated (NFIL3), mRNA. |
| *KLF9* | 230636_s_at | 0.0095458 | 0.373122 | -2 | Hs.150557 | 9q13 | Kruppel-like factor 9 (KLF9), mRNA. |
| *BCL2L11* | 225606_at | 0.000973 | 0.3409764 | -2.5 | Hs.469658 | 2q13 | BCL2-like 11 (apoptosis facilitator) (BCL2L11), transcript variant 6, mRNA. |
| *NFKBIZ* | 223218_s_at | 0.0011736 | 0.357325 | -2.5 | Hs.319171 | 3p12-q12 | nuclear factor of kappa light polypeptide gene enhancer in B-cells inhibitor, zeta (NFKBIZ), transcript variant 2, mRNA. |
| *LUC7L* | 1557067_s_at | 0.0020606 | 0.357325 | -2.5 | Hs.16803 | 16p13.3 | LUC7-like (S. cerevisiae) |
| *IRAK2* | 231779_at | 0.0025569 | 0.357325 | -2.5 | Hs.449207 | 3p25.3 | interleukin-1 receptor-associated kinase 2 (IRAK2), mRNA. |
| *LOC731149* | 213310_at | 0.002646 | 0.357325 | -2.5 |  | 8 | PREDICTED: hypothetical protein LOC731149 (LOC731149), mRNA. |
| *SOCS3* | 227697_at | 0.0030365 | 0.357325 | -2.5 | Hs.527973 | 17q25.3 | suppressor of cytokine signaling 3 (SOCS3), mRNA. |
| *CD44* | 229221_at | 0.003262 | 0.357325 | -2.5 | Hs.502328 | 11p13 | CD44 molecule (Indian blood group) |
| *CXCL1* | 204470_at | 0.0035384 | 0.357325 | -2.5 | Hs.789 | 4q21 | chemokine (C-X-C motif) ligand 1 (melanoma growth stimulating activity, alpha) (CXCL1), mRNA. |
| *FLNA* | 213746_s_at | 0.0037992 | 0.357325 | -2.5 | Hs.195464 | Xq28 | Filamin A, alpha (actin binding protein 280) |
| *NKX3-1* | 209706_at | 0.003835 | 0.357325 | -2.5 | Hs.55999 | 8p21 | NK3 transcription factor related, locus 1 (Drosophila) (NKX3-1), mRNA. |
| *PTGER4* | 204897_at | 0.0042544 | 0.357325 | -2.5 | Hs.199248 | 5p13.1 | prostaglandin E receptor 4 (subtype EP4) (PTGER4), mRNA. |
| *NFKB1* | 209239_at | 0.0042565 | 0.357325 | -2.5 | Hs.654408 | 4q24 | nuclear factor of kappa light polypeptide gene enhancer in B-cells 1 (p105) (NFKB1), mRNA. |
| *PARD6B* | 235165_at | 0.0048958 | 0.357325 | -2.5 | Hs.589848 | 20q13.13 | par-6 partitioning defective 6 homolog beta (C. elegans) (PARD6B), mRNA. |
| *JUN* | 201465_s_at | 0.0049795 | 0.357325 | -2.5 | Hs.525704 | 1p32-p31 | jun oncogene (JUN), mRNA. |
| *SLC22A1* | 207201_s_at | 0.0051139 | 0.357325 | -2.5 | Hs.117367 | 6q26 | solute carrier family 22 (organic cation transporter), member 1 (SLC22A1), transcript variant 1, mRNA. |
| *TRIM36* | 219736_at | 0.0051207 | 0.357325 | -2.5 | Hs.519514 | 5q22.3 | tripartite motif-containing 36 (TRIM36), transcript variant 1, mRNA. |
| *MAP3K8* | 205027_s_at | 0.0051416 | 0.357325 | -2.5 | Hs.432453 | 10p11.23 | mitogen-activated protein kinase kinase kinase 8 (MAP3K8), mRNA. |
| *KCNJ2* | 206765_at | 0.0052416 | 0.357325 | -2.5 | Hs.1547 | 17q23.1-q24.2 | potassium inwardly-rectifying channel, subfamily J, member 2 (KCNJ2), mRNA. |
| *AMPD3* | 207992_s_at | 0.0052596 | 0.357325 | -2.5 | Hs.501890 | 11p15 | adenosine monophosphate deaminase (isoform E) (AMPD3), transcript variant 2, mRNA. |
| *CD83* | 204440_at | 0.0060488 | 0.357325 | -2.5 | Hs.654558 | 6p23 | CD83 molecule (CD83), transcript variant 2, mRNA. |
| *CDC42EP3* | 209288_s_at | 0.0062956 | 0.357325 | -2.5 | Hs.369574 | 2p21 | CDC42 effector protein (Rho GTPase binding) 3 (CDC42EP3), mRNA. |
| *IBRDC2* | 228153_at | 0.0068917 | 0.357325 | -2.5 | Hs.148741 | 6p22.3 | IBR domain containing 2 (IBRDC2), mRNA. |
| *SLC25A37* | 221920_s_at | 0.0076926 | 0.357325 | -2.5 | Hs.122514 | 8p21.2 | solute carrier family 25, member 37 (SLC25A37), mRNA. |
| *C6orf141* | 1554314_at | 0.0077573 | 0.357325 | -2.5 | Hs.485528 | 6p12.3 | Chromosome 6 open reading frame 141 |
| *SLC25A37* | 242335_at | 0.0078932 | 0.357325 | -2.5 | Hs.122514 | 8p21.2 | solute carrier family 25, member 37 (SLC25A37), mRNA. |
| *YPEL2* | 227020_at | 0.0083747 | 0.357325 | -2.5 | Hs.463613 | 17q22 | yippee-like 2 (Drosophila) (YPEL2), mRNA. |
| *PMAIP1* | 204285_s_at | 0.0086491 | 0.3587368 | -2.5 | Hs.96 | 18q21.32 | phorbol-12-myristate-13-acetate-induced protein 1 (PMAIP1), mRNA. |
| *SLC25A37* | 226179_at | 0.0002069 | 0.233361 | -3.3333333 | Hs.596025 | 8p21.2 | Solute carrier family 25, member 37 |
| *NFKBIZ* | 223217_s_at | 0.0002939 | 0.2454125 | -3.3333333 | Hs.319171 | 3p12-q12 | nuclear factor of kappa light polypeptide gene enhancer in B-cells inhibitor, zeta (NFKBIZ), transcript variant 2, mRNA. |
| *LOC286109* | 1561042_at | 0.0008453 | 0.3119901 | -3.3333333 | Hs.660189 | 8q24.3 | Hypothetical protein LOC286109 |
| *ELAVL2* | 228260_at | 0.0023528 | 0.357325 | -3.3333333 | Hs.166109 | 9p21 | ELAV (embryonic lethal, abnormal vision, Drosophila)-like 2 (Hu antigen B) (ELAVL2), mRNA. |
| *ARL5B* | 242727_at | 0.0032482 | 0.357325 | -3.3333333 | Hs.25362 | 10p12.33 | ADP-ribosylation factor-like 5B (ARL5B), mRNA. |
| *TUBB2B* | 214023_x_at | 0.0052731 | 0.357325 | -3.3333333 | Hs.300701 | 6p25 | Tubulin, beta 2B |
| *DAGLB* | 225833_at | 0.0054754 | 0.357325 | -3.3333333 | Hs.487498 | 7p22.1 | diacylglycerol lipase, beta (DAGLB), mRNA. |
| *GEM* | 204472_at | 0.005674 | 0.357325 | -3.3333333 | Hs.654463 | 8q13-q21 | GTP binding protein overexpressed in skeletal muscle (GEM), transcript variant 2, mRNA. |
| *TNFAIP8* | 210260_s_at | 0.0059345 | 0.357325 | -3.3333333 | Hs.656274 | 5q23.1 | tumor necrosis factor, alpha-induced protein 8 (TNFAIP8), transcript variant 2, mRNA. |
| *FAM110C* | 226863_at | 0.0061139 | 0.357325 | -3.3333333 | Hs.8379 | 2p25.3 | Family with sequence similarity 110, member C |
| *CXCL3* | 207850_at | 0.0072672 | 0.357325 | -3.3333333 | Hs.89690 | 4q21 | chemokine (C-X-C motif) ligand 3 (CXCL3), mRNA. |
| *MYLIP* | 223130_s_at | 0.0073454 | 0.357325 | -3.3333333 | Hs.484738 | 6p23-p22.3 | myosin regulatory light chain interacting protein (MYLIP), mRNA. |
| *ACRC* | 238825_at | 0.0017121 | 0.357325 | -5 | Hs.135167 | Xq13.1 | acidic repeat containing (ACRC), mRNA. |
| *TNFAIP3* | 202643_s_at | 0.0074935 | 0.357325 | -5 | Hs.591338 | 6q23 | tumor necrosis factor, alpha-induced protein 3 (TNFAIP3), mRNA. |
| *NR4A2* | 216248_s_at | 0.0082576 | 0.357325 | -5 | Hs.563344 | 2q22-q23 | nuclear receptor subfamily 4, group A, member 2 (NR4A2), transcript variant 4, mRNA. |
| *CXCL2* | 209774_x_at | 0.0099216 | 0.3735558 | -5 | Hs.590921 | 4q21 | chemokine (C-X-C motif) ligand 2 (CXCL2), mRNA. |
